# Meniscal allograft transplantation after meniscectomy: clinical effectiveness and cost-effectiveness

**DOI:** 10.1007/s00167-019-05504-4

**Published:** 2019-04-13

**Authors:** Norman Waugh, Hema Mistry, Andrew Metcalfe, Emma Loveman, Jill Colquitt, Pamela Royle, Nick A. Smith, Tim Spalding

**Affiliations:** 1 Division of Health Sciences, Warwick Medical School, Gibbet Hill Campus, University of Warwick, Coventry, CV4 7AL UK; 2 Warwick Clinical Trials Unit, University of Warwick, Coventry, UK; 3 Effective Evidence, Waterlooville, Hampshire UK; 4 Department of Orthopaedics, University Hospitals Coventry and Warwickshire, Coventry, UK

**Keywords:** Meniscal allograft transplantation, Cost-effectiveness

## Abstract

**Purpose:**

To assess the clinical effectiveness and cost-effectiveness of meniscal allograft transplantation (MAT) after meniscal injury and subsequent meniscectomy.

**Methods:**

Systematic review of clinical effectiveness and cost-effectiveness analysis.

**Results:**

There is considerable evidence from observational studies, of improvement in symptoms after meniscal allograft transplantation, but we found only one small pilot trial with a randomised comparison with a control group that received non-surgical care. MAT has not yet been proven to be chondroprotective. Cost-effectiveness analysis is not possible due to a lack of data on the effectiveness of MAT compared to non-surgical care.

**Conclusion:**

The benefits of MAT include symptomatic relief and restoration of at least some previous activities, which will be reflected in utility values and hence in quality-adjusted life years, and in the longer term, prevention or delay of osteoarthritis, and avoidance or postponement of some knee replacements, with resulting savings. It is likely to be cost-effective, but this cannot be proven on the basis of present evidence.

**Level of evidence:**

IV.

**Electronic supplementary material:**

The online version of this article (10.1007/s00167-019-05504-4) contains supplementary material, which is available to authorized users.

## Introduction

The meniscal cartilages spread the weight-bearing forces in the knee, thereby preserving the articular cartilage on femoral condyles and tibial plateau. The lateral meniscus is thought to carry approximately 70% of the load in its compartment, and the medial one 50%, when the leg is straight. Hannon et al. [18] and the IMREF 2015 Consensus statement [16] have provided reviews of the history of meniscectomy and of meniscal allograft transplantation (MAT).

Meniscal injury and subsequent meniscectomy is thought to lead to early osteoarthritis (OA) because of increased stress on the central articular cartilage. The articular cartilage under the menisci is thinner than on other parts of the tibial plateau [56] and so the sub-meniscal region is more at risk of OA if the meniscus is removed. Because acute meniscal injuries often occur in sport, those afflicted are often young. For example, in the case series of 63 patients reported by Cameron and Saha [10] the average age at meniscectomy was 24. An even younger cohort was reported by Pengas et al. (from the 1960s and 1970s) [36] in which 313 patients with mean age 16 (range 10–19) at meniscectomy were followed up for about 40 years (mean age at assessment 57, range 43–67). OA was found in 87% of meniscectomised knees but in only 18% of non-operated knees. All were either symptomatic (mean KOOS 70) or (13%) had had knee replacement.

Acute meniscal injuries due to trauma in young people should be distinguished from the degenerative meniscal lesions that are common in older people—25% in age range 50–59 years, increasing to 45% in those aged 70–79. The ESSKA 2016 consensus [7] was that meniscectomy should not be a first-line treatment in degenerative meniscal lesions.

Several authors have asserted that meniscectomy leads to OA [4, 13, 21], but the evidence is mixed for several reasons. One is duration of follow-up. Jackson [20] reviewed 577 knees after meniscectomy and compared them with the patients’ other knees. Definite radiographic degenerative changes were much more common in meniscectomised knees (21% versus 5%) but took time to develop, being seen in 22% (control knees 4%) at under 20 years, 53% at 20–29 years (controls 13%) and 67% at 30–40 years. However, only about half of those with radiological degeneration had painful knees.

Given what is known about the functions of the meniscus, meniscectomy would be expected to increase the risk of osteoarthritis (OA). However, for assessing the cost-effectiveness of MAT in reducing OA, we need to know the risks of OA after meniscectomy with and without MAT.

The systematic review by Smith et al. [46] concluded that in 35 studies with mean follow-up 5.9 years, there was good evidence that MAT improved symptoms after meniscectomy, but that there was insufficient evidence as to whether MAT would be chondroprotective. Smith et al. noted the lack of randomised controlled trials (RCTs) of MAT versus conservative care. It would be much easier to quantify the effect of MAT on OA if data were available on matched patients that did not have MAT.

One problem, as Ahn et al. [4] have pointed out, is that patients having meniscectomy may have sustained articular damage in the same event that led to meniscal injury. So OA in a meniscectomised patient may not be due entirely to the absence of a meniscus. However, in the study by Roos et al. [40], 19% had evidence of articular cartilage damage at the time of meniscectomy but 71% had OA in the knees 21 years later, with 48% having Kellgren and Lawrence (KL) grades of 2 or worse. Roos et al. compared the prevalence of OA in meniscectomised knees to those in a population-based control group, in which any OA was seen in 18% and KL grade 2 or worse in 7%. Unfortunately the response rate amongst the invited controls was under 40%, and those who responded may have had more knee pathology, and hence interest, than those who did not. Roos et al. make a useful point about using contralateral knees in meniscectomised patients for control purposes, because the other knees had higher rates of OA than the control group, relative risk (RR) 1.5 for any OA. So it could be argued that using contralateral knees as controls may under-estimate the effect of meniscectomy. Conversely, if the contralateral knees have an increased risk of OA, some of the OA seen in the meniscectomised knee may not be due to the meniscectomy. It is known that OA in one knee causes ‘overloading’ of the other side (that is, OA in the contralateral knee may be blamed on OA in the meniscectomised joint), but not the degree to which this is important, over and above a person’s pre-determined genetic tendency to OA [30].

Another issue is how OA is determined—radiological or symptomatic. In a study of elite American football players with mean age 23 by Smith et al. [45], OA was defined as moderate-to-severe non-focal articular cartilage loss on MRI or joint space narrowing on plain radiographs. They were selected for imaging because they had had previous surgery or had knee symptoms. OA was seen in 15%, but in 4% of those with no previous knee surgery and in 27% of those who had had partial meniscectomy. Mean BMI was 32. Their symptom scores were not reported, but all were still functioning at a high level.

Paradowski et al. [34] also assessed OA by radiology, defining it as joint space narrowing or the presence of osteophytes, equivalent to KL stage 2, in cohorts from 1973, 1978 and 1983–1985. Follow-up varied in duration, as did the type of meniscectomy, with total or subtotal in the early years, but with more (35%) partial meniscectomy in later years. Mean age at meniscectomy was 35 years, and at follow-up 60 (range 34–85) years. Radiographs of the other knees were also taken. The prevalence of tibio-femoral OA at last follow-up was 68% in the meniscectomised group and 36% in the other knees (some of which may have had other surgery).

Claes et al. [12] carried out a meta-analysis of 16 studies of OA after anterior cruciate ligament (ACL) reconstruction, with a minimum follow-up of 10 years, published before October 2010. 11 studies with 614 patients were used for analysis of the effect of meniscectomy. There was considerable heterogeneity amongst studies, but this was in effect size not direction. The overall OR for OA after meniscectomy was 3.5 [95% confidence interval (CI) 2.6–4.9], with OA seen in 50% after meniscectomy and in 16% in control knees.

Other variables that affect the incidence of OA include which cartilage was removed, and whether there is varus or valgus mal-alignment. Allen et al. [5] followed up 230 patients who could be traced out of a series of 428 who had meniscectomy in the years 1958–1970. Some had died but 210 (49% of the original cohort) were reviewed 10–22 years post-meniscectomy. Age at meniscectomy ranged from 13 to 67 years and at follow-up from 29 to 85 years. Radiographs were obtained of both knees. Over half the meniscectomised knees were clinically normal at mean follow-up of 17 years, but radiological signs of OA were seen in 18% of meniscectomised knees compared to 5% of the control knees. OA was more frequent after lateral meniscectomy than medial, presumably because the lateral meniscus covers more of the tibial plateau. After lateral meniscectomy, OA was more common in valgus knees, and after medial meniscectomy, OA was more common in varus knees.

The lateral meniscus is normally crescent shaped but is occasionally larger, even a complete circle. This is known as a discoid cartilage, and may be more prone to injury. Ramme et al. [37] compared the cost-effectiveness of MAT and partial meniscectomy in young women with torn discoid lateral meniscus and concluded that MAT was cost-effective.

Meniscectomy can be total or partial. Andersson-Molina et al. [6] noted radiographic changes 14 years after meniscectomy, including joint space narrowing in 13 of 18 patients after total meniscectomy, but only 6 of 18 after partial meniscectomy. Joint space narrowing was seen in seven patients after total meniscectomy but in only one who had partial meniscectomy. Andersson-Molina et al. [6] compared the meniscectomised group with 36 matched controls (from a local football club) with no history of knee injury, none of whom had joint space narrowing. Despite the radiographic changes, about 70% of the meniscectomy group had normal Lysholm scores at 14 years.

Rongen et al. [39], using data from the Osteoarthritis Initiative, found that meniscectomy conferred a hazard ratio for OA of 3.03 compared to a matched group that had not had meniscectomy. The meniscectomised group (335 patients) had a higher rate of total knee replacement (TKA) than the controls, 18% versus 11%. However, this study is perhaps not as relevant to this review as some others, because the patients studied were in age range 45–79, so many would have had degenerative change in their menisci rather than acute trauma.

A key question is quantifying the risk of OA after meniscectomy compared to knees that do not have meniscectomy. Table 1 shows some relative risk ratios at different intervals with fair consistency in the relative risks ranging from 3.1 to 5.8.

**Table 1:** Risk of osteoarthritis after meniscectomy

	OA after meniscectomy	OA no meniscectomy	Relative risks
Duration of follow-up			
10–19 years			
Allen et al. [5]	18%	5%	3.6
Jackson et al. [20]	23%	4%	5.8
20–29 years			
Jackson et al.	53%	13%	4.1
40 years			
Pengas et al. [36]	87%	16%	4.8
>10 years			
Claes et al. [12]	50	16	3.1

The IMREF 2015 Consensus statement [16] concluded that meniscectomy increases the risk of OA, and recommended three main indications for MAT:

- Unicompartmental pain following total or defunctioning subtotal meniscectomy.
- As a concomitant procedure to ACL reconstruction in order to prolong the life of ACL reconstruction.
- As a concomitant procedure to articular cartilage repair in a meniscus-deficient compartment.

However, the IMREF consensus recommended that MAT was not indicated in patients with no meniscus but no symptoms. This could be seen as a problem given that people may be developing OA without symptoms in the early stages. The decision was based on a paucity of evidence of chondroprotective benefit in asymptomatic people, and consideration of the significant re-operation rate after MAT (as high as 35%).

Some authorities recommend MAT only in knees with little or no degenerative change [56].

The aim of this study was to assess the cost-effectiveness of MAT after meniscectomy. The benefits of MAT could be, firstly relief of symptoms and restoration of quality of life, and secondly, avoidance or delay in the development of symptomatic osteoarthritis and the subsequent need for knee replacement.

## Materials and methods

A systematic review of clinical effectiveness was carried out. A number of recent systematic reviews were identified, their quality assessed, and their conclusions summarised. A table of studies included in these reviews was created. Their inclusions varied because their topics of interest varied. A search for recent studies not included in those reviews was carried out to update them. Greater weight was given to prospective studies.

Fuller details of methods are given in the full report on the ESSKA website, but in brief;

- The databases Ovid Medline, Ovid Embase, Web of Science and the Cochrane Library were searched for articles published from the year 2000 until February 15th 2018. The Medline search strategy and the numbers of records obtained are shown in Appendix 1 to the Supplementary files.
- Titles and abstracts of retrieved studies were screened by two people, with full papers obtained if inclusion or exclusion was uncertain from the abstract.
- Standard systematic review methods were used with quality assessment of included studies using standard checklists for both reviews and primary studies, and checking of data extractions by a second reviewer.

Data were sought on:

- Quality of life, preferably using a generic preference-based measure or a condition-specific measure that could be mapped to a utility measure such as EQ-5D.
- Data on failures and reoperations and graft survival.
- The development of OA, and the frequency of knee replacement, either unicompartmental or total.
- Data on costs, both short-term (the costs of MAT), medium term (the cost of treatment of both those having MAT and those having non-operative care) and long-term (the costs of OA and arthroplasty).

All included studies were assessed for methodological quality using recommended criteria. The Cochrane risk of bias (ROB) assessment criteria [19] were to be used for RCTs and controlled clinical trials (CCTs). Six possible biases were assessed: selection bias, performance bias, detection bias, attrition bias, reporting bias and other bias. For non-randomised studies, tools developed by the National Institute for Health, National Heart, Lung and Blood Institute (NIH NHLBI) [31] re used. These tools focus on biases (selection, performance, detection and attrition), confounding, power and strengths of associations between treatments and outcomes. The tools for cohort studies cover two group comparisons, before and after studies (one group) and case series studies (one group, no before measure).

## Results

Thirty-seven papers from 19 studies of MAT were included, the main papers being references [1, 2, 11, 13, 17, 22, 24, 28, 29, 32, 33, 35, 38, 42, 43, 48, 49, 51, 52, 54] (full list of articles in supplementary file).

There was a range of study designs (Supplementary file, Table 1), with one cohort study (with comparator group but the comparison was not relevant to the review), 12 single arm before and after studies (which report variables at both baseline and follow-up), and six case series. Study characteristics and baseline characteristics of the participants are summarised in the Supplementary file, Table 2. Definitions of failure and periods of follow-up varied, and are summarised in Supplementary file Tables 2 and 3. Mainly because of the observational design, the quality of these non-randomised studies was mostly graded fair, with only two studies [28, 29] rated as good, because they reported intervention and results more clearly, and two studies rated as poor due to insufficient reporting of selection criteria, high loss to follow-up and lack of blinding of outcome assessors [11, 24] and lack of statistical comparison before and after the intervention [11]. Interpretation of some studies was complicated by other procedures, for example in the study by Saltzman et al. study [43] all patients in the full thickness defect arm had cartilage repair procedures, mainly osteochondral allograft. A publication from the study by Saltzman et al. [44] reported outcomes for 40 patients undergoing concomitant MAT and anterior cruciate ligament reconstruction. Where reported, around 20–60% of people underwent concomitant procedures. Further details are provided in the Supplementary file, Table 2.

Cohort size in the included studies ranged from 30 to 313, with a total of 1731 people undergoing at least one MAT. Twelve of the studies were conducted in the USA. Average follow-up in the studies ranged from 2.5 years to 17.3 years, although there was a large variation in the ranges. The key indications for MAT or study inclusion criteria are given in Supplementary file Table 2, but the usual indication was persistent symptoms after meniscectomy. Some studies (such as [13]) included only patients with relatively well-preserved articular cartilage, or well-aligned knees [17], but others included people with more evidence of advanced OA [43, 49] partly to assess survival of MAT in the knees with OA. Marcacci et al. [28] required the other knee to be healthy. Rue et al. [42] included people undergoing simultaneous combined MAT and cartilage repair procedures including autologous chondrocyte implantation (ACI) or fresh osteochondral allograft (OCA) transplantation, in the same compartment. Abrams et al. [2, 3] included people undergoing combined MAT and OCA transplant. A proportion of the people in the remaining studies underwent a variety of concomitant procedures (around 31–73%); these are summarised in Supplementary file Table 2. Mean age mostly ranged from 25 to 45 years, although in the study by Stone et al. [48] the mean age was 47 years. The study by Riboh et al. [38] included only adolescents, age range 13–16 years. The proportion of men in the studies ranged from 48 to 80%, with the majority of studies having at least 60% men, except for 28% in the study of adolescents. [38].

### Failure and survival

The definitions of failure used in the studies differed, and included need for revision surgery (MAT or TKA), removal of graft, and persistent pain (Supplementary file Table 3).

Graft failure, as shown in Table 2, ranged from 3.6% in the bony-fixation subgroup of Abat et al. [1] (success defined as patient satisfaction at 5 years follow-up) to 29% in the studies by Van der Wal (13.8 years of follow-up) [53]. Kempshall et al. [22] found that when failure occurred it was early, potentially indicating a problem with healing or integration of the graft, and reported a mean time to failure of 1.12 years (range 0.47–1.85; SD 0.55). Time to failure ranged from mean 5.2 years in the study by Stone et al. [49] to 10.3 years in the one by Van der Wal [53]. These rates reflect the baseline state of the knees, with the patients in the Stone et al. study having significant OA.

**Table 2:** Failure rates in MAT studies

Study	Number of MATs	Mean FU (range) years	Defn failure	Proportion failed
Abat et al. [1], González-Lucena et al. [17]	88	5 years (2.5–10)	Removal of graft	Suture only 9%
	Suture fixation 33			Bone fixation 3.6%
	Bony fixation 55			
Cole et al. [13]	40	2.8 years (2–4.8)	Conversion to KA	7.5%
Kim et al. [23]	110	4.1 years (2–13.7)	Poor clinical results	10.9%
			Failure (resection, TKA, poor Lysholm)	2% 10 year, 7% 15 years
Marcacci et al. [28]	32	3.4 years (3–5.5)	Debridement, meniscectomy or poor result	6.3%
Mahmoud et al. [27]	45	8.6 years (SD 3.4)	Removal of MAT or KA	OCS 0–2 no failures
				OCS 3–4 26%
McCormick et al. [29]	200	4.9 years (2–9.8)	Revision MAT or TKA	4.7%
				1.5% conversion to KR
Noyes et al. [33]	40	3.3 years (2.0–5.8)		7.9%
Noyes and Barber-Westin [32]	58	17.3 years	Persistent pain or detached or torn allograft	15.3%
Parkinson et al. [35]	124	3 years (1–10)	Graft removal, revision MAT or KA	10.5%
Riboh et al. [38]	32	7.2 years (2–15)	Not reported (but revision MAT was an outcome	22%
Rue et al. [42]	31	3.1 years	Revision or removal	6%
Saltzman et al. [43]	ND 22	ND 4.5 years	Revision MAT or	ND 15%
	FTD 69	FTD 2.5 years	KA	FTD 16.2%
	MAT + ACL 40	MAT + ACL 5.7 years		MAT + ACL 20%
Stone et al. [48]	119	5.8 years (0.2–12.3)	Removal of the allograft without revision, removal and new MAT, or KA	20.1%
Stone et al. [49]	49	8.6 years (2–15)	KA, removal of MAT, pain greater than pre-operatively, or constant moderate pain with no relief from non-operative treatment	22.4%
Van der Wal et al. [53]	63	13.8 years (SD 2.8)	Persistent pain, unsuccessful KASS, poor Lysholm score, detached allograft (2002); removal of MAT, UKA or TKA (2009)	29%
Van der Straeten et al. [52]	329	6.8 (0.2–24.3)	Removal of MAT, KA	27.4%
				19% to KR
Verdonk et al. [54]	100	7.2 years (0.5–14.5)	HSS pain subscore < 30, HSS function score < 80, KR	Medial 28%, lateral 16%

*HSS* Hospital for special surgery, *KASS* knee assessment scoring system, *TKA* total knee arthroplasty, *UKA* unicompartmental knee arthroplasty, *ND* no chondral defect, *FTD* full thickness chondral defect

The results of the Kempshall et al. study [22] show that MAT gives better results in terms of failure rates, if done before the articular cartilage is advanced to the stage of bare bone, with 2-year survival 98% amongst those with articular cartilage in good condition, versus 78% in those with poor condition, but even then it was beneficial with similar absolute increases in clinical scores, though from different baselines. The more advanced group had more concomitant procedures (about 80% compared to 35% in the good cartilage group) with the difference mainly in microfracture.

From the same group, Parkinson et al. [35] reported the results of a series of 125 MATs over a 10-year period, with median follow-up 3 years but range 1–10 years. Those with ICRS grade 3b changes (full thickness articular cartilage loss on either femoral condyle or tibial plateau or both) had much higher failure rates 5 years after MAT. The Kaplan–Meier survival curves show most failures (defined as a need for removal or revision of the MAT or knee replacement) occurred in the first 4–5 years. Survival was better with lateral than with medial allografts. The mean age at meniscectomy was 24 and at MAT 31 years (range 8–49 years). However, even in the worst group with bare bone on both femur and tibia, the 5-year MAT survival was 62%. Most failures occurred in the first few years.

The proportions having reoperations, including revision MAT, debridement or removal of the allograft, varied amongst studies, partly because of duration of follow-up. In their study of 329 MAT implants, Van der Straeten et al. [52] reported that 27% of allografts were removed after a mean time in situ of 8.5 years. In the study of 200 patients by McCormick et al. [29], 32% had subsequent surgery by a mean follow-up 6 years, with mean time to re-operation 21 (range 2–107) months. Abat et al. [1] and González-Lucena et al. [17] reported that 21.4% and 7.3% of the suture-only (by 6.5 years) and bony-fixation groups (by 5 years), respectively, underwent revision surgery involving refixation or removal of the allograft. Carter et al. [11] found 17.5% had had partial meniscectomy by 10 years of follow-up. Noyes and Barber-Westin [32] reported that during 15 years follow-up of 69 patients, half required reoperation relating to failure of the transplant (removal 15, revised 6, TKA 10, osteotomy 2, unicompartmental knee arthroplasty (UKR) 4). In the study in adolescents by Riboh and colleagues [38] only two of 32 MATs required meniscal re-operation after mean follow-up of 7 years.

Repeat MATs were performed in 2% of cases by van der Straeten et al. [52], in 6% by McCormick et al. [29], in 10% (4 of 40 MATs) by Noyes and Barber-Westin [32], and in 10% (5 of 49) by Stone et al. [49], and 12% in the study by Saltzman et al. [43]. Three studies reported no repeat MATs [1, 11, 38].

Conversions to TKA or UKA varied with duration of follow-up, being reported in 3% of cases in the study by Saltzman et al. [43] after a mean follow-up of 4 years, 6% in the study by McCormick et al. [29] by 6 years, 7% in the study by Cole et al. [13] with mean follow-up 33 months, 19% in the Van Der Straeten et al. study [52] at a mean of 11.5 years, and 25% by 15 years in the study by Noyes and Barber-Westin. [32].

Nine studies reported Kaplan–Meier survival analysis at various time points as shown in Table 3. 5-year survival rates were very good, but varied amongst group according to the state of chondral surfaces at baseline, as shown in the group with full thickness chondral loss on both condyles in the study by Parkinson et al. [35]. Ten-year survival ranged from 45% in the “worst case” results from Noyes and Barber-Westin [32] to over 90% in two studies [23, 29], but most studies reported survival in the 60–75% range. However, as Noyes and Barber-Westin [32] report, some of the failures in their worst-case scenario were found only on radiographic or MRI imaging, in patients with no symptoms.

**Table 3:** Long-term survival of MAT

Study	Survival at 5-year time periods			
	5 years	10 years	15 years	20 years
Kim et al. [23] subgroup with ≥ 8 years follow-up (%)		98	93	
Parkinson et al. [35]				
Group 1 (%)	97			
Group 2 (%)	82			
Group 3 (%)	62			
Mahmoud [27]	92% (from graph)	75% (from graph)		
McCormick et al. [29]	95%^a^	93% (from graph)		
Noyes and Barber-Westin [32]				
Worst case (%)	77	45	19	
Clinical failures (%)	84	64	50	
Saltzman et al. MAT + ACL [44] (%)	84	45		
Van der Wal et al. [53]	95% (from graph)	67% (76% lateral, 56% medial)	53%	
Van der Straeten et al. [52]	80%	75%	50%	15% at 24 years
Verdonk et al. [54]			At 14 years	
Lateral MATs (*n *= 61) (%)	90	70	70	
Medial MATs (*n *= 39) (%)	86	74	53	
Medial MATs with high tibial osteotomy (*n *= 13) (%)	100	83	83	

Noyes and Barber-Westin [32] worst case includes some patients with no symptoms related to the transplant but who have MRI grade-3 signal intensity, major extrusion or a tear, signs of a meniscal tear on clinical examination; or radiographic complete loss of joint space. Clinical failures include transplant removal or revision, total or unicompartmental knee replacement, osteotomy, or pain with daily activities

Figures for Stone and Van der Straeten 5, 10 and 15 years, taken from KM graphs and are approximate

Parkinson et al. Baseline data. Group 1 intact articular cartilage or partial thickness loss. Group 2 full thickness loss on one condyle. Group 3 full thickness loss on both condyles. Kim defined failure defined as resection of graft, conversion to THA, or Lysholm score < 45 or less than before MAT

^a^McCormick—but 32% had subsequent surgery usually debridement

15-year survival ranged from 19 to 93%, but the 19% was in the Noyes and Barber-Westin worst-case scenario, whereas the clinical survival rate was 50% [32]. Most studies reported about half of the MATs surviving at 15 years.

Van der Straeten et al. [52] reported 15.1% survival at 24 years, with a mean allograft survival time of 15.2 years.

So for economic analysis, it might be reasonable to assume around 20% have repeat surgery in the short-term but, based on the 15-year follow-up in the study by Noyes and Barber-Westin [32], around half in the longer term, but that clinical success rate at 15 years seems good compared to the natural history in untreated meniscectomised patients.

### Progression of osteoarthritis

Carter et al. [11] reported that at 2 years, 5.9% had mild progression, by 10 years 44.1% had mild progression, 14.7% had moderate-to-advanced progression, and 41.2% had no change. Unfortunately, this study was reported only as an abstract. It reports that plain radiographs were obtained at 2 and 10 years, but not whether progression was based only on radiography. Some X-ray progression is asymptomatic, as reported by Noyes and Barber-Westin [32]. It might be reasonable to assume that the 14.7% would need TKA but that the 44% with mild OA would get by with analgesics. The 14.7% is similar to the results of the 15-year follow-up by Noyes and Barber-Westin [32].

Verdonk et al. [57] reviewed 17 studies of radiological progression. Most studies were short-term (under 5 years) and showed no significant progress but the four studies with longer follow-up (10 years or longer) reported joint space narrowing in 48%, 67%, 75% and all patients.

The systematic review by Smith et al. [46] concluded that there was some evidence that MAT reduces progression of OA, but most studies had short durations of follow-up, the longest being a study by Verdonk et al. [55] with 12 years follow-up, though in that study 11 of the 41 patients also had tibial osteotomy.

In their study of 313 participants, van der Straeten et al. [52] reported progression of osteoarthritis as measured by the KL scale. At a mean of 6.8 years 40% of participants had no progression, 35% progressed more than grade 1, 20% more than two grades and 5% more than three grades.

Ahn et al. [4] reported the result of 69 MATs carried out by a single surgeon from 2005 to 2012. OA was defined by KL grade changes. Patients having any concomitant surgery were excluded, as were patients with OA worse than KL grade 2. Most (about 80%) had KL 0 or 1. At 3-year follow-up, progression of OA was seen in 31 patients at average follow-up 5.5 years, but not in 38, mean FU 4 years. They noted that progression was more frequent after medial meniscectomy than lateral, with medial:lateral odds ratio 3.4 (95% CI 1.2–9.3, *p* = 0.018). However, there were no differences in Lysholm or Tegner scores between the groups. Ahn et al. [4] concluded that it was possible that MAT reduced the risk of OA but that RCTs were necessary.

### Functional outcomes

Table 4 shows results for functional outcomes. Nine studies reported Lysholm scores, all showing good results, with most reporting improvements of over 20 points. Eight reported International Knee Documentation Committee (IKDC) scores, with improvements ranging from 14.8 to 29.8, with most studies reporting gains of over 20 points. Five studies showed improvements in Tegner scores.

**Table 4:** Functional outcomes after MAT

Study (and number of initial patients)	Baseline value (SD)	Endpoint value (SD)	Change (note 1)	*p* value
Lysholm				
Abat et al. [1] *n* = 88	65.4 (11.6)	88.6 (7.2)	23.1 (NR)	< 0.001
Carter et al. [11] *n *= 40	47 (32–68)	71 (38–95)	24 (NR)	
Cole et al. [13] *n *= 36	52.4 (20.26)	71.6 (19.7)	19.2 (NR)	< 0.05
Kim et al. [23] modified Lysholm (*n *= 110)	73.2 (10.6)	89.4 (13.2)	16.2 (NR)	< 0.001
Marcacci et al. [28] *n *= 32	59.8 (18.3)	84.8 (14.4)	25 (NR)	< 0.0001
Saltzman et al. [43]				
ND (*n *= 22)	41.5 (22.3)	NR	14.8 (14.4)^a^	NR
FTD (69)	43.4 (17.4)	NR	21.1 (19.8)^a^	NR
Saltzman et al. [44] MAT + ACL (40)	44 (16)	67 (22)	23 (NR)	< 0.01
Riboh et al. [38]	43.80 (20.37), *n *= 30	58.52 (17.92), *n *= 23	14.4 (NR)	= 0.03
Rue et al. [42] *n *= 28	48.7 (16.4)	74.0 (17.7)	25.3 (NR)	< 0.001
Van der Wal et al. [53] *n *= 49	36.36 (18)	61.06 (20)	24.7 (NR)	0.001

	Baseline value (SD or 95% CI)	Endpoint value (SD or 95% CI)	Change (SD)	*p* value
IKDC				
Carter et al. [11] *n *= 40	50.6 (32.2–68.9)	70.1 (39.1–93.1)	19.5 (NR)	
LaPrade et al. [24] *n *= 40	54.5	72.0 (*n *= 34)	17.5 (NR)	< 0.001
Marcacci et al. [28] *n *= 32	47.4 (20.6)	77.2 (15.6)	29.8 (NR)	< 0.0001
Rue et al. [42] *n *= 28	38.7 (12.7)	66.9 (17.2)	28.2 (NR)	< 0.001
Saltzman et al. [43]				
ND, *n *= 22	41.5 (22.3)	NR	14.8 (14.4)	NR
FTD, *n *= 69	43.4 (17.4)	NR	21.1 (19.8)	NR
Saltzman [44] Concomitant MAT + ACL *n *= 40	44 (16)	67 (22)	23 (NR)	< 0.01
Riboh et al. [38] *n *= 32 (subset of Cole study)	40.2(19) *n *= 27	65.0 (17.7) *n *= 23	24.8 (NR)	< 0.0001
Stone et al. [48] *n *= 115	45 (*n *= 66)	70 (*n *= 15)	25 (NR)	< 0.001
Stone et al. [49] *n *= 49	Median^b^ 48	75	27 (NR)	0.001

	Baseline	Endpoint	*p* value
Tegner			
Cole et al. [13]	5.0 (2.8)	6.5 (2.7)	< 0.05
González-Lucena et al. [17]	3.1 (2.1)	5.5 (2.1)	< 0.001
Marcacci et al. [28]	3 (IQR 3.5)	5 (IQR3–6)	0.012
Rue et al. [42]	5.0 (3.3)	6.7 (2.3)	0.001
Stone et al. [49] (medians)	2.8	5.2	n.s.

Note 1: Where papers have not reported changes, these have been calculated but SDs, SEs and CIs are not available

*NR* not reported by study authors

The studies by Noyes et al. [33] and Noyes and Barber-Westin [32], using the Cincinnati Knee Rating System (CKRS), reported statistically significant improvements in pain, swelling, perception, walking and stairs, but not in the knee giving way. LaPrade et al. [24] found a highly statistically significant improvement in the overall CKRS, from 55 to 73.

Five studies [13, 22, 38, 42, 44] reported Knee Injury and Osteoarthritis Outcome Score (KOOS), and found statistically significant improvements in all subscales, except for the pain subscale in one small study in an adolescent population [38]. Another only reported the endpoint values and is of limited value [53]. In the study by Saltzmann et al. [44], the improvement exceeded the minimally clinically important differences on the symptoms and quality of life subscales in two subgroups that reported this, and in pain and sport in the full-thickness chondral defects subgroup.

Cole et al. [13] and Rue et al. [42] also reported statistically significant differences in the Noyes symptom and sports activity scores.

The Western Ontario and McMaster Universities Arthritis Index (WOMAC) was used in three studies [38, 44, 48] with some significant improvements in the function and total scores (Supplementary file Table 6). Stone et al. [48, 50] found a significant (*p* < 0.001) improvement in overall score and pain after 10 years.

Kempshall et al. [22] reported outcomes by baseline articular cartilage state (Table 5) showing that end results were poorer in patients with more severe baseline articular cartilage damage than in those with good chondral surface states., but they started with poorer scores and the actual improvements were of similar magnitude, for example with improvements in Lysholm scores of 21.6 in the good baseline group (58.6–80.2) and 24 in the poor group (47.3–71.4), when grafts survived.

**Table 5:** Functional outcomes by baseline joint state

	Chondral surface good, *n *= 60	Chondral surface bare *n *= 39
Kempshall et al. [22]		
Lysholm knee score at final endpoint, mean (SD)^a^		
Baseline value	58.6 (4.8)	47.3 (6.6)
Endpoint value	80.2 (5.0)	71.4 (7.8)
*p* value	< 0.001	< 0.001
IKDC score at final endpoint, mean (SD)		
Baseline value	43.13 (4.1)	37.3 (5.3)
Endpoint value	68.8 (5.5)	58.7 (8.2)
*p* value	< 0.001	< 0.001
Tegner score at final endpoint, median (range)		
Baseline value	2 (0–7)	2 (0–9)
Endpoint value	4 (1–10)	4 (1–9)
*p* value	< 0.05	< 0.05
Mahmoud et al. [27]	OCS 0–2	OCS 3–4
Lysholm knee score		
Change from baseline	+ 21.3	+24.5
*p* value	0.013	< 0.001
IKDC		
Change from baseline	10.8	21.5
*p* value	0.241	0.001
Tegner		
Change from baseline	1.73	0.53
*p* value	0.015	n.s.

*OCS* Outerbridge Cartilage Score

Mahmoud et al. [27] also noted greater improvements in Lysholm and IKDC scores in those with more advanced chondral damage at baseline (Table 4).

Lee et al. [25] also report benefit from MAT in patients with advanced bipolar chondral lesions, with no difference in post-operative Lysholm scores between those with baseline ICRS scores ≤ 2 and those with more advanced chondral damage (baseline data not provided for subgroups). Clinical failure rates were not significantly different (low grade damage 7%, high grade 5%, but there were small numbers and wide CIs), but graft survival rates by MRI or follow-up arthroscopy showed a much lower survival rate in the group with high-grade lesions on both sides at baseline (62%) compared to those with low-grade lesions (94%).

One study each reported the Knee Society Score (KSS) [23] and Hospital for Special Surgery (HSS) score [54] and reported statistically significant improvements.

### Quality of life

Six studies [2, 13, 28, 38, 42, 44] (Table 6) reported QoL using Short-Form 36 (SF36) or the shorter subset, SF-12. One [44] found no significant differences Cole et al. [13] and Riboh et al. [38] found improvements in the physical but not mental scores, as did Abrams et al. [2] in a group having combined MAT and osteochondral allografts. Rue et al. [42] found a statistically significant improvement in the physical SF-12 only in the subgroup that had combined MAT and autologous chondrocyte implantation. Both physical and mental components were significantly improved in the study by Marcacci et al. [28] which was rated highly, based on appropriate selection of participants, sample size calculation, blinding of outcome assessors and no losses to follow-up.

**Table 6:** Quality of life in MAT studies

Outcomes, mean (SD)	Kempshall et al. [22]^a^		Cole et al. [13]	Marcacci [28]	Rue [42]	Abrams et al. [3]	Riboh [38]
	Chondral surface good *n *= 60	Chondral surface bare *n *= 39	*n *= 36	*n *= 32	*n *= 28	*n *= 32	
KOOS QoL							
Baseline value	28.9 (5.0)	22.4 (5.0)	26^b^		25.2 (18.9)		
Endpoint value	52.7 (7.1)	45.0 (8.1)	50^b^		55.1 (20.4)		
*p* value	*p *< 0.001	*p *< 0.001	*p *= 0.16		<0.001		
SF-12/36 physical			SF-36	SF-36	SF-12		
Baseline value			40^b^	37.3 (7.2)	38.9 (7.3)		38.6 (6.6)
Endpoint value			48^b^	49.7 (8.3)	44.0 (5.5)		46.6 (6.8)
*p* value			*p *< 0.05	*p *< 0.0001	0.001		
SF-12/36 mental			SF-36	SF-36	SF-12		SF12
Baseline value			50^b^	49.7 (10.8)	55.5 (9.4)		54.0 (11.7)
Endpoint value			55^b^	53.5 (7.5)	55.2 (8.2)		55.8 (8.0)
Change value			NR	NR	*p *< 0.34		
*p* value			*p *= ns	*p *= 0.0032			
SF-12 overall							
Baseline value						43.5 (5.6)	
Endpoint value						46.6 (5.9)	
*p* value						0.041	

^a^Outcomes assessed at 2 years, not mean follow-up of 2.9 years, results described as mean (95% CI)

^b^Estimated from figure

### Subgroups: medial vs lateral MAT

Nine studies [1, 13, 17, 24, 28, 42, 43, 51, 54] assessed medial and lateral MATs separately (Supplementary file, Table 7) for at least one functional or quality of life outcome, but only one [43] found statistically significant differences in functional outcomes or quality of life between medial and lateral MAT, in a group of patients having combined MAT and anterior cruciate ligament reconstruction, with only seven patients in the lateral group. In an earlier paper from the same group, Cole et al. [13] commented that the lateral subgroup showed a trend toward greater improvements than the medial subgroup on nearly all knee scoring scales.

Eight studies [23, 32, 33, 35, 43, 48, 53, 54] reported survival outcomes for medial and lateral MAT, six showing no statistically significant differences. Parkinson et al. [35] found increased graft survival with lateral MAT (89% at 5 years versus 62% for medial, *p* = 0.026) (Supplementary File Table 8). Kim et al. [23] reported 13% of failures in lateral MAT compared to 3.7% in medial MAT, but the medial figure was based on only one patient, and some patients were classed as failures because of MRI findings despite satisfactory Lysholm scores. Van der Wal et al. [53] did not present statistical analysis, but failure rates were higher in the medial subgroup (35%) than the lateral (25%) and time to failures was shorter (mean 82 months medial versus 161 months lateral). Verdonk et al. [54] reported 28% failures in medial MAT compared to 16% in lateral MAT, but the difference was not statistically significant.

### Subgroups: combined procedures

Seven studies considered concurrent procedures [13, 17, 24, 28, 32, 33, 42] (see also supplementary file Tables 9 and 10). Most studies found no statistically significant differences between those undergoing isolated MAT or MAT combined with other concurrent procedures on either functional, quality of life or survival estimates. However, Noyes et al. [32] found a statistically significantly poorer survival time in knees that required a concurrent osteochondral autograft transfer, compared to those that did not. Rue et al. [42] noted differences in IKDC score between those undergoing MAT + ACI and MAT + OA transplantation, however there was also an imbalance in scores at baseline. Noyes et al. [33] also reported analysis of those having ligament reconstruction or osteochondral autografts, and those having MAT alone, and found no significant differences.

### Meniscectomy versus no meniscectomy

Li et al. [26] compared two groups of meniscectomised patients, one group having MAT and the other not. About half of the MAT group had allografts inserted at the time of meniscectomy. The rest had MAT on average 3 years later because of pain in the knee. At average follow-up of 54 months (minimum 40 months), clinical results were similar, but there was less radiographic change in the MAT group. The study was too small and too short duration to compare immediate versus delayed MAT.

### Return to sport

Saltzman et al. [44] and Stone et al. [49] reported return to sport. In the subgroup having concomitant MAT and ACL reconstruction in the Saltzman et al. study [44], 19 participants self-identified as athletes and 50% of these returned to sports, 39% at the same level as previously. Of 10 who had been involved in competitive sport, 50% returned to sport at the same level. This study was assessed as fair quality, although note that the sample for these outcomes was small. Stone et al. [49] reported that 74% of the 49 participants in their study were able to participate in sport.

### Adverse events

Complications were generally infrequent. Van Arkel et al. [51] reported no major complications and only five minor ones amongst 63 grafts, these being irritation around non-absorbable sutures. Three studies reported a need to resolve flexion problems by manipulation or arthrolysis a few weeks after MAT in four of 38 patients [33], one of 32 patients [28] and one of 32 patients [38]. Stone et al. [48] reported four infections amongst 115 patients, and Saltzman et al. [44] reported two minor infections in 40 patients. LaPrade et al. [24] reported one late infection but it appears unrelated.

Kempshall et al. [22] reported major complications in 17% of the ‘chondral surface good’ group and 38% of the ‘chondral surface bare’ group, this included tear of the allograft in 13% and 31%, respectively. Abat at al [1] reported total complication rates of 33.3% of the only-suture group and 16.4% of the bony-fixation group. Three studies reported that no complications occurred [2, 13, 42] and in one study adverse events were not reported [11].

#### Assessing the cost-effectiveness of meniscal allograft transplantation

The benefits of MAT could include symptomatic relief and restoration of at least some previous activities, which will be reflected in utility values, and in the longer term, prevention or delay of osteoarthritis, and avoidance or postponement of some knee replacements, with resulting savings.

The costs include the initial procedure, the cost of the allograft, and any subsequent surgery, including arthroscopic debridement if the graft has to be removed, and possibly insertion of a second MAT.

For cost-effectiveness analysis, data are needed for both patients having MAT, and a comparison group that does not have MAT after meniscectomy, as follow:

- Quality of life expressed as a utility measure using a generic preference-based measure such as EQ-5D-5L, or a clinical outcome score such as WOMAC, from which we can map to EQ-5D. This captures symptomatic relief and return to activities. (Though EQ-5D may not capture all the utility of return to sport since it is based more on activities of daily living.) Increasing OA would reduce quality of life over time.
- Costs of MAT including rehabilitation.
- Costs of MAT failure, including if appropriate, repeat MAT. A second MAT in the same compartment is uncommon [29, 32, 43, 49, 52]. However if a first MAT in a young person lasted for, say, 10 years, providing symptomatic relief, there could be a case for a repeat MAT, perhaps as an interim intervention pending knee arthroplasty.
- Costs of conservative care for people not having MAT, including physiotherapy and drug costs. In this case, “physiotherapy” would need to be carefully defined, since it is an umbrella term covering many different forms of treatment, and a personalised knee therapy intervention for the meniscal deficient knee may be more effective. (See pilot RCT by Smith et al. [47]).
- Costs of advanced OA, principally TKA, for both groups. This will depend on proportions having TKA.

However, data are lacking on:

- What proportion of people who do not get MAT, develop advanced OA requiring TKA, and when. It is assumed that surgeons will be reluctant to do TKA before age 55.
- What proportion of people who do get MAT, develop advanced OA requiring TKA, and when.

Almost all the studies of MAT are observational ones with no non-MAT control groups. However Smith et al. [47] have reported the results of a pilot RCT of MAT versus personalised physiotherapy. At 12 months, KOOS scores improved in both groups but the improvement in the MAT group was roughly double that in the physiotherapy group—a difference of 12 in composite KOOS (*p* = 0.03). Other scores improved more in the MAT group but without reaching statistical significance. Smith et al. advocate caution in interpretation due to small numbers (21 randomised, plus a preference group of 15), the short follow-up, and possible effects of the three osteotomies in the MAT group but none in the physiotherapy group. They use the data to estimate that a trial with 50 patients in each arm would be required to give a definitive result.

## Discussion

There seems to be no doubt that meniscectomy, whether total or partial, leads to OA in the longer term. However, it is not yet proven whether MAT is chondroprotective. With the data currently available, it does not appear possible to do a full assessment of the cost-effectiveness of MAT. The main problem is the lack of control groups, having active conservative care. MAT is probably better, but for cost-effectiveness analysis we need to know the effect size—how much better is it?

The people who get significant problems after meniscectomy may be a small subset, perhaps only 10–20% of all having meniscectomy. Those who present with significant symptoms with or without radiological evidence of OA at 5–10 years after meniscectomy, may have other risk factors. It is assumed that people having MAT have had a traumatic meniscal tear and subsequent meniscectomy, and that they do not come from the older group with degenerate menisci, whose natural history is different (and who should not be used as a natural history comparison group).

To compare outcomes of MAT and non-MAT, data are needed on how people are selected for MAT in order to identify a comparable group of people in non-MAT natural history studies. In some MAT studies, patients with more severe knee problems such as existing OA, or advanced KL grades, are excluded. So some non-MAT people may have a more severe mix of knee problems. If so, comparing a MAT cohort with people who do not get MAT after meniscectomy, may favour MAT. But more likely, if the MAT group are a small subset of all people who have had meniscectomy, and who are doing worse, then a comparison with a non-MAT group may under-estimate the benefits of MAT.

The likelihood of success is greater in those with less damage to the articular cartilage. A particularly difficult group are young people with ICRS grade 3 lesions in whom conservative treatment has failed, and who are much too young for knee replacement. Two options have been tried. One is the “biological knee replacement” combining MAT with ACI as reported by Bhosale et al. [9] from Oswestry, but with a series of only eight patients. The other is to combine MAT with osteochondral allografts, as reported by Abrams et al. [2] and Frank et al. [15].

However, the results of the study by Kempshall et al. and Parkinson et al. [22, 35] show that in patients with more severe “bone on bone” defects, MAT may not be as successful as in people with lesser defects, but can still provide benefit. It is possible that MAT may be both less successful and more cost-effective in the more severe group because they have more to gain. The gains in symptom scores were of similar magnitude but the severe group started from a lower baseline.

A short-term before and after analysis could compare quality of life using an instrument that can be converted to a utility measure, such as WOMAC or SF-12, and assessing the cost per quality-adjusted life year (QALY) gained from the quality of life gains. However, the improvements may be due to MAT, or associated rehabilitation, or some natural recovery, or by patients learning to live with the problem, for example by reducing activities. Data are lacking data on what benefits the non-MAT measures might provide for non-MAT patients. They would be expected to benefit from physiotherapy, if they get it. But if the MAT group got physiotherapy and the non-MAT group did not, it would not be clear whether any benefits were due to MAT or physiotherapy.

So a before and after analysis could be misleading, and favourable to MAT. However the pilot RCT by Smith et al. [47] reported that MAT had advantages over conservative care with personalised physiotherapy.

A study by Bendich et al. [8] has addressed this problem. They noted that there was uncertainty about the chondroprotective effect of MAT [41, 46, 52]. They then asked how effective MAT would have to be, to be cost-effective. They start by assuming that after meniscectomy, progression to severe OA (bad enough for TKA to be considered) would be 1.8% a year, based on the study by Englund et al. [14], but they then do sensitivity analyses around that figure, with higher and lower progression rates. In their primary “base case” analysis, patients are aged 30, with no OA, and BMI 20. They test various other scenarios, with older or heavier patients, and different ages at which TKA would be performed. They also test different costs of MAT, TKA and of non-operative care.

In their base case, they estimate that MAT would have to reduce progression to severe OA by 31%, from 1.8% a year to 1.2% a year. However in patients with higher BMIs, who are at increased risk of progression to OA, the reductions need only be 16% in the BMI 25–30 group, and 10% in the BMI over 30 group, for MAT to be cost-effective. Their base case age was 30. In patients aged 20–29 (who would have to wait much longer for TKA), the reduction in progression for MAT to be cost-effective was only 25%, whereas in the 40–49 age group, the reduction in progression would need to be 41%.

The costs used were from the USA, including TKA cost of $26,452, and only slightly higher for revision TKA. Costs in other countries would be different. They assumed cost of MAT to be $8202.

In their base case, they assumed no OA, whereas we know that many people who have needed meniscectomy have sustained articular cartilage damage. In those people, progression to OA would be faster, and cost-effectiveness of MAT greater. They do not give details of interval between meniscectomy and MAT. Their benefits focus on reducing progression to OA and joint replacement, rather than on relief of symptoms after meniscectomy.

The pilot RCT by Smith et al. [47] showed greater benefit with MAT than personalised physiotherapy and it may be that MAT could be shown to be cost-effective based on utility gain from symptom relief alone, with taking into account future cost of TKA avoided. A similar but much larger and longer trial is needed to confirm this.

As outlined above, there appears to be a subgroup that do particularly badly after meniscal tears and subsequent meniscectomy. MAT would be much more cost-effective in this group, so a high research priority is how to identify them at or soon after meniscectomy, and to see if intervention soon after meniscectomy gave better result than waiting for symptoms to develop.

Research priorities include the need for randomised controlled trial evidence for the effectiveness of MAT compared with conservative care in short-term effectiveness, and also the long-term potential to change the natural history after meniscal loss. Further research is also warranted on the best choice of graft, surgical technique, and optimal rehabilitation after surgery.

## Conclusions

There were three issues to be considered:

1. Does meniscal deficiency lead to early osteoarthritis? Yes.
2. Does MAT prevent or delay OA after meniscectomy? There is a lack of good evidence on this, so the verdict at present is “not proven”.
3. Is MAT an effective and cost-effective way of relieving continuing symptoms after meniscectomy? It appears effective, but the effectiveness of MAT compared to conservative management is uncertain, with only the one small RCT providing short-term evidence.

## Electronic supplementary material

Below is the link to the electronic supplementary material.

Supplementary material 1 (DOCX 223 kb)
