# Supplementary material for: Meniscal allograft transplantation after meniscectomy: clinical effectiveness and cost-effectiveness
Source: Knee Surg Sports Traumatol Arthrosc. 2019 Apr 13;27(6):1825–39. doi: 10.1007/s00167-019-05504-4 (PMC6541576; doi:10.1007/s00167-019-05504-4)
Supplement: Supplementary file 1 — Supplementary material 1 (DOCX 223 kb) [file 167_2019_5504_MOESM1_ESM.docx]

**Knee Surgery Sports Traumatology Arthroscopy**

Supplementary files to Waugh et al. KSST-D-18-01629

# Title: Meniscal allograft transplantation after meniscectomy: clinical effectiveness and cost-effectiveness

AUTHORS: Norman Waugh (MRCP (UK)^1, 5^, MPH), Hema Mistry (PhD)^1^, Andrew Metcalfe (FRCS, PhD)^2^, Emma Loveman (PhD)^3^, Jill Colquitt (PhD^3^, Pamela Royle (PhD)^1^, Nick A Smith (FRCS, PhD)^4^, Tim Spalding (FRCS)^4^

AUTHOR AFFILIATIONS

^1^ Division of Health Sciences, Warwick Medical School, University of Warwick, Coventry UK

^2^ Warwick Clinical Trials Unit, University of Warwick, Coventry, UK

^3^ Effective Evidence, Waterlooville, Hampshire, UK

^4^ Department of Orthopaedics, University Hospitals Coventry and Warwickshire, Coventry, UK

^5^ Corresponding author

Electronic address: [norman.waugh@warwick.ac.uk](mailto:norman.waugh@warwick.ac.uk)

Contents

[Title: Meniscal allograft transplantation after meniscectomy: clinical effectiveness and cost-effectiveness 1](#_Toc4154385)

[Table S1: MAT studies included in this review 3](#_Toc4154386)

[Table S2. MAT studies characteristics and participant baseline characteristics 4](#_Toc4154387)

[Table S3. Definitions of failure after MAT 10](#_Toc4154388)

[Table S4: Failure rates MAT studies 12](#_Toc4154389)

[Table S5: Time to failure, revision and conversion in MAT studies 12](#_Toc4154390)

[Table S6: Key functional and quality of life outcomes after MAT 13](#_Toc4154391)

[Table S7: Medial and lateral MAT subgroups: functional outcomes^a^ and quality of life 16](#_Toc4154392)

[Table S8a: Medial and lateral MAT subgroups: survival, prospective studies 19](#_Toc4154393)

[Table S8b: Medial and lateral MAT subgroups: failure, survival and reoperations, retrospective studies 19](#_Toc4154394)

[Table S9: Combined procedures subgroups: functional outcomes^a^ and quality of life 21](#_Toc4154395)

[Table S10: Combined procedures subgroups: survival 22](#_Toc4154396)

[References 23](#_Toc4154397)

[Appendix 1: Literature searches 26](#_Toc4154398)

## Table S1: MAT studies included in this review

| **Author** | **Intervention details** | **Study Design** |
| --- | --- | --- |
|  | | |
| Abat et al [1] and Gonzales-Lucena et al [6] | MAT (2 groups different fixation methods) then sub-study of suture only group | Cohort |
| Carter et al [4] (abstract only) | MAT | Before and after |
| Cole et al [5] and Abrams et al [3] [2] | MAT, with subgroup having OCA | Before and after |
| Kim et al [9] [8] [10] [12] [13] | MAT | Retrospective before and after study |
| LaPrade et al [11] | MAT | Before and after |
| Mahmoud [14] | MAT | Prospective case series |
| Noyes and Barber-Westin [17] | MAT | Before and after |
| Noyes et al [19, 20] | MAT | Before and after |
| Marcacci et al [15] | MAT | Before and after |
| McCormick et al [16] | MAT | Case series |
| Riboh et al [22] adolescent group | MAT | Before and after |
| Rue et al [23] | MAT and OA transplant or MAT and ACI | Before and after |
| Parkinson et al [21] Kempshall et al [7] | MAT, with groups according to articular cartilage condition | Case series |
| Riboh et al [22] adolescent group | MAT | Before and after |
| Saltzman et al [26] [25] [24] | MAT (+ Subgroup MAT+ACL) | Before and after |
| Stone et al [27] [30] [28] [29] | MAT | Case series |
| Riboh et al [22] adolescent group | MAT | Before and after |
| Van Arkel et al[31] [32] and Van der Wal et al [34] | MAT | Before and after study |
| Van Der Straeten et al [33] | MAT | Case series |
| Verdonk et al[35] [36] | MAT | Before and after |

## Table S2. MAT studies characteristics and participant baseline characteristics

| **Study** | **Indication / inclusion criteria** | **Concomitant procedures** | **Baselines** |
| --- | --- | --- | --- |
| **Barcelona** | | | |
| **Abat et al [1]**  **Country:** Spain  **Study design^a^:** Prospective cohort study  **Follow-up:** 5 years (range 2.5–10)  **Sample size:** 88 (only-suture 33, bony-fixation 55) | Two studies of different ways of securing MAT, sutures or bone plugs in patients with joint pain due to a previous total or subtotal meniscectomy (postmeniscectomy syndrome), 2001 to 2008. 88 patients. 2012 paper reports extrusion rates. 2013 paper reports function and radiographic appearance | 39% of only-suture group, 43% of bony fixation group, including ACL reconstruction, microfracture, chondral shaving, hardware removal, arthroscopic cartilage repair with TruFit plugs. | **Age^a^ :** 37.3 (15-51)  **% male:** 64  **Location^b^:** 45/55 |
| **González-Lucena et al [6]**  **Country:** Spain  **Study design^a^:** Before and after  **Follow-up**: 78 months (range 63-96)  **Sample size:** 33 | This paper report the subgroup of 33 patients from the above study who did not have bone plugs. 2001-2013 | 39%: ACL reconstruction, microfracture, chondral shaving | **Age^a^ :** 38.8 (21-54)  **% male:** 72.7  **Location^b^:** 42.4 / 57.6 |
|  | | | |
| **Carter et al [4]** (abstract only)  **Country:** USA  **Study design^a^:** Before and after  **Follow-up duration**: 10 years  **Sample size:** 40 (41 allografts) | Inclusion criteria not reported | 73%: ACL, osteotomy, ACL/osteotomy, medial lateral ligament. | **Age^a^ :** 34.8 (19-50)  **% male:** NR  **Location^b^:** NR |
| **Rush Centre, Chicago, USA** | | | |
| **Cole et al [5]**  **Country:** USA  **Study design^a^:** Before and after  (author design: case series)  **Follow-up**: 33.5 months (range 24-57).  **Sample size:** 40 (45 transplants) | Persistent symptoms after meniscectomy, relatively well-preserved articular cartilage with less than grade III changes on radiographs and at arthroscopy, normal knee alignment, and a stable joint. Joints that could be rendered stable or realigned by a concomitant procedure at the time of transplantation were also included. Minimum follow-up 24 months. 1997-2003. | 47.5% osteochondral allografts, osteochondral allografts, osteochondral autografts, microfracture, osteochondritis dissecans fixations, autologous chondrocyte implantation, chondral debridement. Also ligament reconstruction and osteotomy. | **Age^a^ :** 31 (SD 9.5)  **% male:** 61.1  **Location^b^:** 62.5/37.5 |
| **Rue [23]**  **Country:** USA  **Study design^a^:** Before and after study (author design: case series)  **Follow-up**: mean 3.1 years (SD 1.2, range 1.9-5.6)  MAT+ACI mean 3.4 (range 1.9-5.6) years  MAT+OA mean 2.9 (range 1.9-5.0) years  **Sample size:** 30 (31 procedures) | Simultaneous combined MAT and cartilage repair procedures including ACI or fresh OCA transplantation, in the same compartment, 1997 – 2004. Inclusion criteria were persistent symptoms after meniscectomy with combined articular cartilage injury, normal alignment or correction to normal alignment, and a stable ligamentous knee examination. Minimum 24 months follow-up. | ACI 16 (52%) or fresh OA transplant 15 (48%). (ACI was chosen for relatively younger patients with superficial defects especially of the patellofemoral joint. OA grafts were chosen for older patients with larger defects of the femoral condyle with associated bone loss). Also proximal tibial osteotomy, hardware removal. | **Age^a^ :** 29.9 (13.9-47.9)  **% male:** 60.0  **Location^b^:** 64.5/35.5  There may be some overlap with Cole 2006[5] because same centre and all operations done by single surgeon, but only 3 patients in Cole 2006 had OCA. Also with Abrams. |
| **Abrams et al [2] [3]**  **Country:** USA  **Study design^a^:** Before and After (author definition case series  **Follow-up:** mean 4.4 years (range 2-11)  **Sample size:** 32 | Persistent symptoms after meniscectomy, an isolated ICRS grade 3 or 4 defect of the femoral condyle, normal alignment or correction to normal alignment, ligamentous stability, and minimum 2-year clinical follow-up. 2003 – 2009. | Series having combined MAT and OCA. A small percentage underwent other concomitant procedures, no further details. | **Age^a^ :** 35.0 (10.0)  **% male:** 53.1  **Location^b^:** 75 / 22 / both 3 |
| **Saltzman et al [25] [26] [24]**  **Study design:** retrospective analysis of prospectively collected data. Various subgroups  **Follow-up:** No chondral defect (ND) 4.48 (SD 2.63) years, Full-thickness chondral defects (FTD) 3.84 (SD 2.47) years[25] [25]  Concomitant ACL reconstruction [26]: mean 5.7 (SD 3.2) years (range 1.7-16.5)[26]  **Sample size:** 91 (of 457 operated), 22 ND, 69 FTD. Group of n=40 (of 53) with concomitant ACL reconstruction [26] | All patients who underwent medial or lateral MAT by a single surgeon 1997 – 2013 (1999 -2014[26]. Inclusion criteria: patients with osteochondritis dissecans; isolated single lesions, multiple lesions or bipolar lesions; and minimum 2 years of follow-up. | ACL reconstruction: ND 38%, FTD 12%; cartilage procedure: ND 0%, FTD 100% (OCA70%, ACI 19%, microfracture 13%, OATS 4%, DeNovo 1%); realignment procedure ND 9%, FTD 10%)  Group with concomitant MAT+ACL reconstruction, n=40 [26] (100%) | **Age^a^ :** ND 26.8 (10.7); FTD 30.4 (10.3); subgroup 30.3 (9.6)  **% male:** ND 63.6 FTD 46.4. Subgroup 53  **Location^b^:** ND 77.3 / 22.7; FTD 56.5 / 43.5. Subgroup^b^ 83 / 17 |
| **Riboh et al [22]** Adolescent subgroup  **Study design:** retrospective analysis of prospectively collected data  **Follow-up duration**: 7.2 years (SD 3.2, range 2 to 15)  **Sample size**: 27 (of 32 enrolled, but 23 in results tables) | Age ≤ 16 years at index procedure; MAT for symptomatic meniscal insufficiency (load-related pain and swelling in the compartment undergoing meniscectomy) for which conservative treatment failed; procedure ≤ 2012; and minimum 2-year clinical follow-up | 47% (31% ACI, 6% ACL reconstruction, 3% ACI biopsy, 3% OATS, 9% OA, 3% high tibial osteotomy) | **Age^a^:** 15.4 (1.04)  **% male:** 28.1  **Location^b^:** 16 / 84 |
| **McCormick et al [16]**  **Study design:** retrospective review of prospectively collected data  **Follow-up duration:** 59 months (range 24-118)  **Sample size**: 200 subsequent surgery  172 at final follow-up | MAT in isolation or in combination with cartilage repair or regeneration techniques and bony realignment procedures, 2003 to 2011 | 60% (37% cartilage procedure, 7% cartilage procedure and osteotomy, 11% ACL reconstruction, 8% osteotomy) | **Age^a^:** 34.3 (10.3)  **% male**: 50  **Location:** 64/36/1 (medial/lateral/both) |
|  |  |  |  |
| **LaPrade et al [11]**  **Country:** Vail Colorado, USA  **Study design^a^:** Case series  **Follow-up**: 2.5 years (range 1.8-4.0)  **Sample size:** 40 | Uni-compartmental knee pain and post-activity effusions after total or near-total meniscectomy in patients with closed physes. Patients either demonstrated ligamentous stability or underwent a concurrent cruciate ligament reconstruction surgery to address their instability. 2003-2006. | 52.5% Revision ACL reconstruction, ACL reconstruction, hardware removal, microfracture of femoral condyle, osteoarticular allograft, distal femoral osteotomy. | **Age^a^ :** 25 (16-42)  **% male:** 67.5  **Location^b^:** 47.5/52.5 |
| **Mahmoud et al [14]**  **Country:** Brisbane, Australia  Study design: consecutive series  Follow-up: mean 8.6 years (SD 3.4 yrs)  Sample size: 45 | Patients with pain after meniscectomy, despite minimum of six months non-operative treatment (physio, knee braces, reduced activities). Exclusions: radiographic or arthroscopic evidence of bone-on-bone articulation. | 42%, ACL, PCL, osteotomy, chondral repair | **Age:** 35  **%male:** 51%  **Location**^b^: 66/34 |
| **Marcacci [15]**  **Country:** Bologna, Italy  **Study design^a^:** Before and after (author description: case series)  **Follow-up**: mean 40.4 (SD 6.90, range 36-66) months  **Sample size:** 32 | Patients with total or subtotal chronic meniscal injuries considered eligible for MAT: unicompartmental knee pain after total or subtotal meniscectomy (meniscus loss greater than 75%), anterior cruciate ligament deficiencies stabilized at the time of the index surgery, age 15-55 years, and contralateral healthy knee. 2005-2009. | 31%: ligament reconstruction surgery for patients with ligamentous instability or osteotomy (details reported). | **Age^a^ :** 35.6 (10.03)  **% male:** 72  **Location^b^:** 50/50 |
| **Noyes and colleagues** |  |  |  |
| **Noyes et al [18, 19]**  **Country:** Cincinnati, USA  **Study design^a^:** Before and after study (author description: case series)  **Follow-up**: average 40 months (range 24-69). 2015 study, all 40 transplants 11.0 (range, 0.2 to 17.7) years; 18 transplants completing long-term evaluation: 13.7 (range 8.4 – 17.3) years.  **Sample size:** 38 (40 transplants) | Prior meniscectomy, ≤ 50 years, clinical symptoms of pain in the tibiofemoral compartment, no radiographic evidence of advanced arthrosis, and ≥2 mm of tibiofemoral joint space on 45° weight-bearing postero-anterior radiographs. 1995 – 2000. | 40% osteochondral autograft transfer; 10.5% knee ligament reconstruction; 18.4% anterior cruciate ligament reconstruction; 2.6% medial collateral ligament reconstruction; 2.6% posterior cruciate ligament reconstruction. | **Age^a^ :** 30 (14-49)  **% male:** 52.6  **Location^b^:** 47.4/47.4/ both 5.3 |
| **Noyes and Barber-Westin [17]**  **Country:** USA  **Study design^a^:** Case series  **Follow-up**: unclear for total, appears to be up to 17.3 years (was 13.1 (3.1) years for those failing not requiring surgery) for survival. For functional outcomes was 11.2 (3.2) years (but 10 [2.6] years for those who required later reoperations and 13.3 [2.9] years for those not requiring further surgery).  **Sample size:** 69 (72 transplants) | Prior meniscectomy, <50 years, pain in the involved compartment, ≥2 mm of retained tibiofemoral joint space on 45° weight bearing posteroanterior (PA) radiographs, no or only minimal bone exposure on tibiofemoral surfaces, normal axial alignment.  1995-2005 | 39% of knees concurrent procedures (osteochondral autograft transfers; knee ligament reconstructions; revision knee ligament reconstructions). | **Age^a^ :** 30 (14-49)  **% male:** 47.8  **Location^b^:** 56.9/43.1 |
| **Spalding and colleagues, Coventry, UK** | | | |
| **Parkinson et al [21]** and **Kempshall et al [7]**  **Country:** Coventry, UK  **Study design^a^:** Prospective cohort  **Follow-up**: 3 (range 1-10) years  **Sample size:** 125 | <50 years, experiencing pain, history of total or subtotal meniscectomy in the same compartment of the knee. 2005 – 2014. Divided into three groups according to state of articular cartilage. | 55.2% had associated procedures Kempshall Group A 35%, Group B (bare bones) 79.5%, including osteotomy, revision ACL, meniscal repair, matrix ACI, microfracture, Trufit plug. | **Age^a^ :** 31.0 (8-49)  **% male:** 68.8  **Location^b^:** 20/80 |
| **The Stone Group, San Francisco** | | | |
| **Stone et al [30]**  **Country:** USA  **Study design^a^:** Case series  **Follow-up**: 5.8 years (2 mo to 12.3 yrs).  **Sample size:** 115 (119 MATs) | Aim: to determine whether MAT will survive in an osteoarthitic knee (Outerbridge grades III and IV).  1997-1999  45 patients. Mean age 48, range 14 to 69 years. | All had other procedures to smooth rough articular cartilage (chondroplasty) and most had more than one.  Failure was removal of MAT or joint replacement. | 89.4% MAT survival with mean failure time at 4.4 years. Highly significant improvements in pain and, activity.  So OA is not a contraindication to MAT. |
| **Stone et al [27]** | 119 MATs with simultaneous articular cartilage repair. Pain in the knee due to irreparable damage to the meniscus, or loss of > 50% of the meniscus. Outerbridge grade III or IV changes in the respective compartment. 1997-2008 Age^a^ : 46.9 (14.1-73.2). Almost half over 50. 70% male  Location^b^: 71.4/28.6 | 92% had at least one additional procedure, with a range of one to nine. These included articular cartilage repair by microfracture 69) and articular cartilage paste grafting (67), medial opening tibial osteotomy (15) and ACL reconstruction (17). | Failure in 25 = removal of MAT (7) or KR (18, TKR 10, UKR 8). Revision with new MAT not counted as failure. |
| **Stone et al [29] [28] [30]**  **Country:** USA  **Study design:** Case series (retrospective review of prospectively collected data)  **Follow-up duration**: mean 8.6 (SD 4.2, range 2-15) years  **Sample size:** 49 (76 in the initial study group [68 in earlier abstract] of 159 in the total MAT population) | Those receiving MAT, previous participation in competitive sports who had a pre-injury Tegner level of ≥8, a desire to participate in sports and Outerbridge (OB) Grade III or Grade IV changes.  Subset of 49 patients having MAT on background of moderate to severe articular cartilage damage (OB grade IV in 41, with aim to assess return to active sports.  Mean follow-up 8.6 years, minimum 2 years after MAT | 12.2% medial opening wedge osteotomy (prior to 2003); 69.4% articular cartilage repair (microfracture alone, articular cartilage paste graft alone, or combined); 8.2% ACL reconstruction. 22.4% had cartilage repair 6 weeks prior to MAT. | **Age (range):** 45.3 (14.1 to 73.2)  **% male:**  73.5  **Location^b^:** 73.5 / 24.5  74% returned to sport with improvements in pain and function, but 11 (22%) of MATs failed at mean of 5.2 years |
| **Bin and colleagues, South Korea** |  |  |  |
| **Kim et al 2017 [8] [9] [10] and Lee et al [12] [13]**  **Study design** retrospective before and after study (author description case series)  **Follow-up:** mean 49.4 (range 24-164) months  **Sample size:** 106 (110 knees) | Knees that underwent MAT in a single hospital (1996 – 2009) and were followed up for a minimum of 2 years. Indications: previous subtotal or total meniscectomy followed by persistent swelling and pain in the involved compartment during activities of daily living | At time of MAT or a separate operation: surgical treatment of ACL tears (20% knees); osteochondritis dissecans (3.6% knees); ACL tear with posterolateral corner injury (0.9% knees) and PCL tear (0.9% knees). | **Age (median range):** 33.1 (16-57)  **% male:** 72.6  **Location^b^:** 74.5 / 25.5 |
|  |  |  |  |
| **Van Arkel et al and van der Wal et al [31] [32] [34]**  **Country:** The Netherlands  **Study design:** Before and After  **Follow-up duration:** 13.8 (SD2.8) years (2009); 60 months (4 to 126) (2002)  **Sample size:** 57 at 60 months; 46 at 13.8 years | Patients younger than 55 years with disabling compartmental osteoarthritis after meniscectomy in first 23, then changed to younger than 45 years with stable normally aligned knees but disabling compartmental osteoarthritis after meniscectomy, 1989 - 1999 | 3.5% ACL repair, no further details | **Age^a^:** 39.4 (6.9)  **% male:** 70.2  **Location:** 29.8/59.6/10.5 (medial/lateral/both) |
| **Van Der Straeten et al [33]**  **Country:**  Belgium  **Study design:** Case series (Retrospective review of prospectively collected data).  **Follow-up duration:** mean 6.8 (range 0.2-24.3) years  **Sample size**: 265 (of 313 enrolled) | <60 years, moderate to severe knee symptoms (pain, swelling, instability) shortly after total meniscectomy or after a failed meniscus replacement with an artificial polyurethane meniscus or a collagen meniscal implant. In 6 the MAT was a replacement after failure. 1989 – 2013 | 35.8% including microfracture, osteochondral autograft transfer system, high tibial osteotomy and ACL reconstruction. | **Age (range):** 33.3 (15-57)  **% male:**  60  **Location^b^:** 36 / 64  N=313 allografts |
| **Verdonk et al, 2005 [35] [36] Country:** Belgium  **Study design:** before and after (retrospective analysis of prospectively collected data)  **Follow-up duration:** mean 7.2 (SD 3.6) years (range 0.5 to 14.5)  **Sample size:** 100 | Moderate-to-severe pain in a younger patient (<50 years) who had undergone a previous total meniscectomy, was not old enough to be considered for a knee joint, moderate-to-severe pain. 1989-2001. | 49% of medial allografts and 20% of lateral allografts, High tibial osteotomy 15%, femoral varus osteotomy 2%, ACL reconstruction 3%, osteochondral plug transfer 4%, microfracture 3%. | **Age^a^:** 35.0 (6.7)  **% male:** 72.9  **Location:** 38.5/59/2  (medial/lateral/both) |

^a^mean (range) unless stated otherwise; ^b^ % medial / lateral

## Table S3. Definitions of failure after MAT

| **Study** | **Definition** |
| --- | --- |
| Abat et al [1] and González-Lucena et al [6] | Complete removal of allograft |
| Cole et al [5] | Required conversion to a unicompartmental or total knee arthroplasty |
| Kempshall et al [7] and Parkinson et al [21] | Complete removal, revision or conversion to joint replacement |
| Kim et al [8] [9] [10] | Poor overall results on MRI, arthroscopy or modified Lysholm; or non-satisfactory overall results |
| Marcacci et al [15] | Developed lack of flexion and underwent arthroscopic arthrolysis or symptomatic posterior horn flap lesion of the graft with no history of trauma, or underwent arthroscopic selective meniscectomy and debridement |
| McCormick [16] | Revision MAT or KA |
| Noyes et al [19] | 2004: Persistent pain or mechanical damage (a detached or torn allograft) |
| Noyes and Barber-Westin [17] | Results reported for:  1) reoperations related to failure of transplant (transplant removal or revision, total knee arthroplasty, unicompartmental knee arthroplasty, or osteotomy)  2 ) MRI failure (grade 3 signal intensity or extrusion >50% of the meniscus transplant width) and/or radiograph failure (or loss of joint space in the involved tibiofemoral compartment on 45 degree weightbearing PA radiographs (IKDC grade D) with no reoperation required. |
| Riboh et al, 2016[22] | Not reported (revision MAT was an outcome however) |
| Rue et al [23] | Revision of either the MAT or cartilage repair procedure or  arthroscopic confirmation of MAT or cartilage repair failure |
| Saltzman et al [25] [24] [26] | Additional ACL reconstruction procedure, revision MAT, or conversion to unicompartmental or tricompartmental total knee arthroplasty (reported separately except in the subgroup paper) |
| Stone et al [30] [27] | Removal of the allograft without revision, or progression to a total or unicompartmental knee replacement (removal and revision with implantation of a new meniscal allograft was counted in subsequent surgical procedures). |
| Stone et al [28] [29] | Progression to knee arthroplasty, surgical removal of the MAT without revision, pain greater than pre-operatively, or constant moderate pain with no relief from non-operative treatment. |
| Van Arkel et al and van der Wal et al [31] [32] [34] | Persistent pain, unsuccessful KASS, poor Lysholm score, detached allograft (2002); complete resection of the graft, with or without placement of unicompartmental knee arthroplasty or TKA(2009) |
| Van Der Straeten et al [33] | Removal of allograft including during conversion to TKA or UKA |
| Verdonk et al [35] [36] | Moderate or severe occasional or persistent pain (HSS pain subscore <30) or poor knee function (HSS function score <80), or conversion to total or unicompartmental knee arthroplasty |

ACL: Anterior Cruciate Ligament; HSS: Hospital for Special Surgery; KASS: Knee Assessment Scoring System; TKA: total knee arthroplasty; UKA: unicompartmental knee arthroplasty

## Table S4: Failure rates MAT studies

|  | **Riboh et al [22]** | **McCormick et al [16]** | **Van Arkel et al[32]** | **Verdonk et al [35]** | **Kim et al [10]** | **Van Der Straeten et al [33]** | **Stone et al [29]** |
| --- | --- | --- | --- | --- | --- | --- | --- |
| **Follow-up, years (SD / range)** | 7.2 (SD 3.2 / 2-15) | 4.92 (2-9.8) | 13.8 (SD2.8) | 7.2 (SD 3.6 / 0.5-14.5) | 4.12 (range 2-13.67) | 6.8 (range 0.2-24.3) | 8.6 (SD 4.2 / 2-15) |
| **No. of transplants** | 32 | 200 | 63 | 100 allografts  96 patients | 110 | 329 | 49 |
| **Failure, %** |  | 4.7 | 29 | 21 allografts  18.8 patients | 10.9^b^  18.2^c^ | 27.4 | 22.4 |
| **Conversion to TKA or UKA, %** |  | 1.5 |  |  |  | 19.2 | 1.5 |
| **Subsequent surgery** | 22^a^ | 32^d^ |  |  |  | 61^e^ | Revision: 32.7^f^ |

^a^revision MAT (0%), debridement (6%). ^b^poor overall result. ^c^non-satisfactory overall results. ^d^ Debridement, scar excision, MUA (19%), treatment of progressive disease, same/different compartment (4.5/1%), meniscal graft repair or debridement of <50% removal (1.5%), revision MAT (2%), second-look arthroscopic surgery (1.5%), total knee replacement (1.5%). ^e^menisectomy (8.2%). ^f^Partial meniscectomy (20.4%), suture repair (8.2%), removal and revision MAT (10.2%)

## Table S5: Time to failure, revision and conversion in MAT studies

| **Mean (SD or range) years** | **Saltzman et al [25]** | | | **Stone et al [29]** | **Van Arkel et al [32]** | **Van Der Straeten et al [33]** | **Verdonk et al [35]** |
| --- | --- | --- | --- | --- | --- | --- | --- |
| **Follow-up, years (SD)** | ND 4.48 (2.63) | FTD 3.84 (2.47) | MAT+ACL 5.7 (3.2) | 8.6 (SD 4.2 / 2-15) | 13.8 (SD2.8) | 6.8 (range 0.2-24.3) | 7.2 (3.6 / 0.5-14.5) |
| **No. of transplants** | 22 | 69 | 40 | 49 | 63 | 329 | 100 |
| **Time to failure** |  |  | 7.3 (4.5) | 5.2 (4.4) | 10.3 (4.25-17.3) | 8.5 (0.17- 24) |  |
| **Time to revision MAT** | 2.31 | 2.69 |  |  |  |  |  |
| **Mean time to TKA or UKA,** | TKA: 6.44 | TKA: 6.19 | TKA: 8.3 (4.0) |  |  | TKA: 11.5 (6.55)  UKA: 6.5 (4.47) | 6.3 (3.6) |
| **Time to allograft removal** |  |  |  |  |  | 3.7 (3.86) |  |

ACL: Anterior Cruciate Ligament; FTD: Full-thickness chondral defects; ND: No chondral defect; TKA: total knee arthroplasty; UKA: unicompartmental knee arthroplasty

## Table S6: Key functional and quality of life outcomes after MAT

| **Outcome, mean (SD or range)** | **Saltzman et al [25]**  **Fair Quality** | | | **Riboh et al [22]**  **Fair Quality** | **Van Arkel et al [32]**  **Fair Quality** | **Verdonk et al [35]**  **Fair Quality** | **Kim et al [10]**  **Fair Quality** | **Stone et al [29]**  **Fair Quality** |
| --- | --- | --- | --- | --- | --- | --- | --- | --- |
|  | **ND, n=22** | **FTD, n= 69** | **Concomitant MAT+ACL n=40** | **N=32** | **N=46 (49 allografts)** | **n=96 (100 allografts)** | **N=106 (110 allografts)** | **N=49** |
| **Lysholm,**  Baseline value  Endpoint value  Change value  P-value | 41.5 (22.3)  NR  14.8 (14.4)^a^  NR | 43.4 (17.4)  NR  21.1 (19.8) ^a^  NR | 44 (16)  67 (22)  NR  <0.01 | 43.80 (20.37), n=30  58.52 (17.92), n=23  NR  p=0.03 | 36.36 (18)  61.06 (20)  NR  0.001 |  | Modified Lysholm  92.3 (7.3)  72.7 (12.8)  NR  <0.001 |  |
| **IKDC**  Baseline value  Endpoint value  Change value  P-value | 34.3 (19.9)  NR  15.3 (14) ^a^  NR | 36.2 (15.3)  NR  24.2 (23.1) ^a^  NR | 37 (14)  60 (22)  NR  <0.01 | 40.19 (18.98), n=27  65.02 (17.70), n=23  NR  p<0.0001 | NR  39.99  NR  NR |  |  | Median^b^  48  75  NR  0.001 |
| **KOOS Pain,** Baseline value  Endpoint value  Change value  P-value | 54.0 (16.3)  NR  13.6 (13.4)  NR | 55.0 (15.5)  NR  17.6 (17.1) ^a^  NR | 59 (17)  74 (21)  NR  <0.01 | 64.19 (23.20), n=28  76.57 (14.91), n=23  NR  p=ns | NR  64.72  NR  NR |  |  |  |
| **KOOS Symptoms,**  Baseline value  Endpoint value  Change value  P-value | 56.4 (25.6)  NR  11.3 (12) ^a^  NR | 52.4 (18.1)  NR  15.2 (19.3) ^a^  NR | 59 (17)  68 (18)  NR  <0.04 | 59.73 (17.83), n=29  72.36 (16.48), n=23  NR  p=0.04 | NR  61.41  NR  NR |  |  |  |
| **KOOS ADL**  Baseline value  Endpoint value  Change value  P-value | 60.7 (25.4)  NR  10.1 (9.4)  NR | 68.3 (18.4)  NR  17.3 (17.4)  NR | 72 (19)  85 (16)  NR  <0.01 | 75.38 (22.35), n=27  90.09 (10.53), n=23  NR  p=0.004 | NR  71.31  NR  NR |  |  |  |
| **KOOS Sport**  Baseline value  Endpoint value  Change value  P-value | 24.3 (21.3)  NR  8.3 (12)  NR | 24.1 (20.0)  NR  28.0 (28.2) ^a^  NR | 24 (18)  48 (28)  NR  <0.01 | 35.19 (22.89), n=27  62.61 (25.04), n=23  NR  p=0.002 | NR  36.09  NR  NR |  |  |  |
| **KOOS QOL** Baseline value  Endpoint value  Change value  P-value | 24 (15.5)  NR  20.5 (22.6) ^a^  NR | 27.1 (17.6)  NR  23.1 (26.6) ^a^  NR | 20 (16)  45 (25)  NR  <0.01 | 26.62 (16.86), n=27  54.89 (22.77), n=23  NR  p=0.003 | NR  39.81  NR  NR |  |  |  |
| **WOMAC Pain**  Baseline value  Endpoint value  Change value  P-value | 7.2 (3.7)  NR  -1.7 (2.1)  NR | 7.1 (3.6)  NR  -2.7 (3.2)  NR | 6.9 (3.5)  4.0 (4.1)  NR  <0.01 | 5.20 (4.91), n=30  3.30 (2.62), n=23  NR  p=ns |  |  |  |  |
| **WOMAC Stiffness**  Baseline value  Endpoint value  Change value  P-value | 3.4 (2)  NR  -0.9 (1.4)  NR | 3.6 (1.8)  NR  -1.1 (2.3)  NR | 3.4 (1.9)  2.8 (2.1)  NR  0.22 | 3.10 (1.97), n=29  1.83 (1.23), n=23  NR  p=ns |  |  |  |  |
| **WOMAC Function**  Baseline value  Endpoint value  Change value  P-value | 26.7 (17.2)  NR  -6.9 (6.4) ^a^  NR | 21.6 (12.5)  NR  -11.8 (11.8) ^a^  NR | 19.0 (13.0)  9.9 (11.0)  NR  <0.01 | 16.74 (15.20), n=27  6.74 (7.16), n=23  NR  p=0.004 |  |  |  |  |
| **WOMAC Total**  Baseline value  Endpoint value  Change value  P-value | 33.8 (18)  NR  -9.9 (9.2)  NR | 32.3 (16.6)  NR  -14.8 (15.4) ^a^  NR | 30.0 (18.0)  17.0 (17.0)  NR  <0.01 |  |  |  |  | **Median^b^**  23  3  NR  <0.001 |
| **SF-12 Physical,** Baseline value  Endpoint value  Change value  P-value | 34.5 (5.6)  NR  5.2 (8.3)  NR | 38.4 (7.6)  NR  2.5 (6.9)  NR | 41.0 (7.4)  43.0 (6.5)  NR  0.28 | 38.56 (6.58), n=29  46.59 (6.77), n=23  NR  p<0.0001 |  |  |  |  |
| **SF-12 Mental**  Baseline value  Endpoint value  Change value  P-value | 50.6 (15.8)  NR  -0.8 (9.7)  NR | 52.6 (10.0)  NR  0.5 (8.7)  NR | 49.0 (13.0)  54.0 (9.6)  NR  0.14 | 54.00 (11.72), n=28  55.79 (8.01), n=23  NR  p=ns |  |  |  |  |
| **Tegner,**  Baseline value  Endpoint value  Change value  P-value | NR | NR | 6.3 (2.3)^c^  4.6 (2.5)  1.7  0.02 |  |  |  |  | **Median^b^**  2.8  5.2  NR  0.032 |
| **KSS pain**  Baseline value  Endpoint value  Change value  P-value |  |  |  |  |  |  | 47.7 (5.0)  32.5 (5.9)  NR  <0.001 |  |
| **Modified HSS pain**  Baseline value  Endpoint value  Change value  P-value |  |  |  |  |  | 13.7 (9.0)  39.4 (13.9)  NR 0.000 |  |  |
| **Modified HSS**  Baseline value  Endpoint value  Change value  P-value |  |  |  |  |  | 60.1 (20.5)  88.6 (21.0)  NR  0.000 |  |  |

^a^Improvement from preop. exceeds minimal clinically important difference.

^b^estimated from figures

MEDIAL AND LATERAL MAT SUBGROUPS

## Table S7: Medial and lateral MAT subgroups: functional outcomes^a^ and quality of life

| **Study** | **Medial** | | | | **Lateral** | | | **P value** |
| --- | --- | --- | --- | --- | --- | --- | --- | --- |
| **Abat et al [1]** | **Only-suture, n=33** | | | | **Bony-fixation, n=55** | | |  |
|  | **Medial n=14** | **Lateral n=19** | | | **Medial n=25** | **Lateral n=30** | |  |
| **Lysholm, mean (SD)**  Endpoint value | 88.4 (7.5) | 89 (9.2) | | | 89.2 (7.4) | 93.2 (6.2) | | p=ns |
| **Tegner, median (range)**  Endpoint value | 6 (3–8) | 6 (3–8) | | | 6 (3–9) | 7 (1–9) | | p=ns |
| **Cole et al [5]** | **Medial, n=25** | | | | **Lateral, n=15** | | |  |
| **Lysholm, mean**  Baseline value  Endpoint value  % change  P-value | 52.11  69.20  32.8  0.001 | | | | 52.77  75.60  43.3  0.013 | | | p>0.05  P>0.05 |
| **IKDC, mean**  Baseline value  Endpoint value  % change  P-value | 45.71  60.62  36.3  0.002 | | | | 46.86  69.55  48.4  0.005 | | | p>0.05  P>0.05 |
| **Tegner, mean**  Baseline value  Endpoint value  % change  P-value | 4.45  5.88  32.1  0.091 | | | | 5.86  7.40  26.3  0.261 | | | p>0.05  P>0.05 |
| **SF-36 Physical, mean**  Baseline value  Endpoint value  % change  P-value | 38.84  46.15  18.8  0.052 | | | | 39.31  52.23  32.29  0.004 | | | p>0.05  P>0.05 |
| **SF-36 Mental, mean**  Baseline value  Endpoint value  % change  P-value | 52.16  55.64  6.7  0.307 | | | | 49.23  55.11  11.9  0.154 | | | p>0.05  P>0.05 |
| **Saltzman et al [26]** | **MAT+ACL reconstruction, n=40** | | | | | | |  |
|  | **Medial n=33** | | | | **Lateral n=7** | | |  |
| IKDC, mean (SD) | 56 (22) | | | | 75 (14) | | | 0.06 |
| KOOS ADL, mean (SD) | 80 (21) | | | | 98 (4) | | | 0.05 |
| KOOS sport, mean (SD) | 44 (28) | | | | 67 (17) | | | 0.07 |
| KOOS QoL mean (SD) | 40 (24) | | | | 70 (14) | | | <0.01 |
| WOMAC function mean (SD) | 11 (11) | | | | 1.3 (2.8) | | | 0.03 |
| WOMAC total, mean (SD) | 20 (16) | | | | 4.8 (6.2) | | | 0.04 |
| **González-Lucena et al [6]** | **Medial MAT, n=14** | | | **Lateral MAT, n=19** | | | **P-value** | |
| **Lysholm, mean (SD)**  Endpoint value | 88.37 (7.5) | | | 89 (9.2) | | | 0.64 | |
| **Tegner, mean (SD)**  Endpoint value | 5 (1.53) | | | 6 (2) | | | >0.99 | |
| **LaPrade et al [11]** | **Medial MAT, n=19** | | | **Lateral MAT, n=21** | | |  | |
| **IKDC subjective scores**  Baseline value  Endpoint value  P-value | 51.2  68.2  <0.001 | | | 57.6  76.6 (n=15)  <0.001 | | |  | |
| **Marcacci et al [15]** | **Medial MAT, n=16** | | | **Lateral MAT, n=16** | | |  | |
| **Lysholm, mean (SD)**  Baseline value  Endpoint value | 59.9 (19.6)  83.3 (13.7) | | | 59.7 (17.4)  86.4 (9.7) | | | p=ns | |
| **IKDC, mean (SD)**  Baseline value  Endpoint value | 48.3 (19.8)  77.1 (18.5) | | | 46.6 (21.9)  77.4 (12.5) | | | p=ns | |
| **Tegner activity level, median (IQR)**  Baseline value  Endpoint value | 3 (2-5)  4 (3-7) | | | 4 (3-4)  5 (4-6) | | | p=ns | |
| **SF-36 PCS, mean (SD)**  Baseline value  Endpoint value | 36.1 (7.8)  49.4 (8.9) | | | 38.5 (7.8)  47.6 (7.4) | | | p=ns | |
| **SF-36 MCS, mean (SD)**  Baseline value  Endpoint value | 56.1 (6.9)  56.6 (5.5) | | | 43.3 (9.8)  50.4 (8.4) | | | p=ns | |
| **Rue et al [23]** | **Medial, n=20**  **(7 MAT+ACI, 13 MAT+OA)** | | | **Lateral, n=11**  **(9 MAT+ACI, 2 MAT+OA)** | | |  | |
| **Lyshom, mean (SD)**  Endpoint value | 83.8 (9.5) | | | 76.0 (13.1) | | |  | |
| **IKDC, mean (SD)**  Endpoint value | 79.7 (11.2) | | | 73.3 (10.3) | | |  | |
| **Tegner, mean (SD)**  Endpoint value | 6.8 (1.2) | | | 7.6 (1.8) | | |  | |
| **SF-36 Physical, mean (SD)**  Endpoint value | 44.8 (4.2) | | | 46.1 (3.0) | | |  | |
| **SF-36 Mental, mean (SD)**  Endpoint value | 52.6 (6.7) | | | 56.3 (6.3) | | |  | |
| **Van Arkel et al [32]** | **Medial n=17** | | **Lateral, n=34** | | | **Both n=6** | |  |
| **Lysholm score, Mean (range)**  Baseline value^b^  Endpoint value  p-value | 44.0 (15-86)  55.36 (23-90)  0.134 | | 37.10 (6-65)  63.9 (21-91)  0.000 | | | 37 (15 to 56)  77 (48 to 99)  NR | |  |
| **KOOS, mean, 13.8 years**  Pain  Symptoms  Function in daily living  Sport and recreation  Quality of life | 52.64 (19-100)  54.09 (29-100)  61.09 (34-100)  23.18 (0-100)  27.36 (0-100) | | 66.70 (22-100)  60.10 (32-96)  72.75 (37-100)  40.00 (0-100)  43.00 (6-100) | | |  | | 0.143  0.448  0.219  0.132  0.127 |
|  |  | | |  | | |  | |
| **Verdonk et al [35]** | **Medial, n=39 allografts** | | | **Lateral, n=61 allografts** | | |  | |
| **Modified HSS pain score, mean (SD),**  Baseline value  Endpoint value  P-value | 11.9 (3.9)  34.2 (17.2)  0.000 | | | 14.8 (9.3)  42.7 (10.3)  0.000 | | |  | |
| **Modified HSS function score, mean (SD),**  Baseline value  Endpoint value  P-value | 58.6 (23.6)  83.7 (25.14)  0.000 | | | 61.1 (18.4)  91.64 (17.4)  0.000 | | |  | |
|  | **Isolated medial MATs (20)** | | | **Isolated lateral MATs(49)** | | |  | |
| **Modified HHS pain score** Mean (SD)  Baseline  Endpoint  P value | 11.6 (7.7)  33.5 (18.6)  P = 0.001 | | | 15.3 (9.4)  42.7 (10.1)  P =0.000 | | |  | |
| **Modified HSS function score** mean (SD)  Baseline  Endpoint  P value | 58.7 (27.0)  83.7 (26.3)  P = 0.014 | | | 61.5 (19.5)  92.6 (15.9)  P = 0.000 | | |  | |
| **Failure** | 35% | | | 18% | | |  | |
| **Time to failure** mean (SD) | 6.8 years (4.6) | | | 4.8 (2.9) | | |  | |
| **Proportion surviving** (rounded)  5 years  10 years  15 years | 84%  72%  27% | | | 91%  67%  67% | | |  | |

^a^For studies [5, 23] reporting Noyes and KOOS scores or Cincinnati knee rating scale[11, 17].

^b^Baselines in earlier publications reported to be lateral: 33 (5 to 73); Medial 39 (15 to 76). No statistically significant differences between lateral and medial.

## Table S8a: Medial and lateral MAT subgroups: survival, prospective studies

| **Noyes and Barber-Westin [17]**  ^1^ | **Medial MAT, n=41 (transplants)** | **Lateral MAT, n=31 (transplants)** | **P-value** |
| --- | --- | --- | --- |
| Survival, %, mean (95% CI)  2 years  5 years  7 years  10 years  15 years | 85 (70, 94)  75 (59, 87)  65 (48, 78)  41 (27, 58)  14 (5, 29) | 84 (65, 94)  80.5 (62, 92)  74 (55, 87)  50 (32, 68)  29 (15, 50) | NS |
| Mean (SD) time to failure requiring reoperation, years | 8.2 (5) | 7.7 (5) |  |
| **Parkinson et al [21]** | **Medial, n=25** | **Lateral, n=100** |  |
| 5 year survival, % | 62 | 89 | 0.026 |
| Lateral vs Medial, HR (95% CI) survival | 0.24 (0.07, 0.84), p=0.03 | |  |
| Failures | 6/25 (24) | 7/99 (7) |  |

| **Stone et al [27] [30]** | **Medial, n=85** | **Lateral, n=34** |  |
| --- | --- | --- | --- |
| K-M overall mean survival | 9.9 years (SD 0.5, 95% CI 9.0 to 10.8, 1.3 to 12.3 years) | 10.2 years (SD 0.8, 95% CI 8.6, 11.7, 2 months to 12.3 years) |  |
|  | medial versus lateral HR 1.11 (p=0.848) | |  |

^1^ Short-term results Noyes et al [19]

## Table S8b: Medial and lateral MAT subgroups: failure, survival and reoperations, retrospective studies

| **Study** | **Medial** | | **Lateral** | | | **P value** |
| --- | --- | --- | --- | --- | --- | --- |
| **Saltzman et al [26]** | **MAT+ACL reconstruction, n=40** | | | | |  |
|  | **Medial n=33** | | **Lateral n=7** | | |  |
| No. of reoperations, mean (SD) | 1.1 (0.8) | | 0 (0) | | | <0.01 |
| Time to reoperation, y, mean (SD) | 3.8 (4.2) | | NA | | | NA |
| Athletes returned to plan, n (%) | 5 (15) | | 4 (57) | | | 0.68 |
| Graft failure, n (%) | 8 (24) | | 0 (0) | | | 0.15 |
| **Van Arkel et al [32]** | **Medial n=17** | **Lateral, n=34** | | | **Both n=6** |  |
| Failure, n (%) 13.8 years | 8 (35) | 10 (25) | | |  |  |
| Time to failure, months, mean (range) | 82 (51-97) | 161 (100-208) | | |  |  |
| *Subgroups at 60 months:* |  |  | | |  |  |
| Cumulative survival rate (worst case and clinical criteria), % (95% CI) | 50 (55, 83) | 76 (82, 92) | | | 67 (58, 94) |  |
| Mean survival time, months | 69 | 111 | | | 89 |  |
| Cumulative allograft survival, % (95% CI) (success rate) | 63 (55, 83) | 88 (85, 92) | | | 67 (58, 94) |  |
| **Verdonk et al [35]** | **Medial, n=39 allografts** | | | **Lateral, n=61 allografts** | |  |
| Failure, n/N (%) | 11/39 (28) | | | 10/61 (16) | |  |
| Time to failure, years, mean (SD) | 6.0 (3.8) | | | 4.8 (2.8) | |  |
| mean cumulative survival time, years mean (95% CI) | 11.6 (10.1, 13.1) | | | 11.6 (10.3, 12.9) | | p=0.733 |
| Cumulative Survival Rate, % (SD)  - 5 years  - 10 years  - 14 years | 86.2 (5.7)  74.2 (7.4)  52.8 (14.4) | | | 90.2 (4.2)  69.8 (9.7)  69.8 (9.7) | |  |
| **Kim et al [10]** | **Medial n=27 knees** | | | **Lateral n=83 knees** | |  |
| Failure, % | 3.7 | | | 13.3 | |  |
| **Stone et al [29]** | **Medial, n=37** | | | **Lateral, n=49** | |  |
| Failure % | 27 | | | 41.7 | | 1.00 |

**COMBINED PROCEDURES**

## Table S9: Combined procedures subgroups: functional outcomes^a^ and quality of life

| **Study** | **Procedures** | | **P-value** |
| --- | --- | --- | --- |
| **Abrams et al [2]** | **Combined MAT and OCA**  Lysholm   - Baseline 41.9 (16.1) - Endpoint 63.6 (24.1 - P <0.001   IKDC   - Baseline 32.9 (11.4) - Endpoint 55.3 (23.6 - P < 0.001   KOOS   - Baseline 42.5 (11.7) - Endpoint 62.7 (21.0) - P < 0.001 |  |  |
| **Cole et al [5]** | **Isolated MAT, n=21** | **Combined, n=19** |  |
| **Lyshom, mean**  Baseline value  Endpoint value  % change  P-value | 47.94  68.05  41.9  0.002 | 57.4  75.53  31.6  0.006 | p>0.05  P>0.05 |
| **IKDC, mean**  Baseline value  Endpoint value  % change  P-value | 43.90  61.77  40.7  0.002 | 48.75  66.46  36.3  0.004 | p>0.05  P>0.05 |
| **Tegner, mean**  Baseline value  Endpoint value  % change  P-value | 5.39  6.14  13.9  0.326 | 4.63  6.83  47.5  0.032 | p>0.05  P>0.05 |
| **SF-36 Physical, mean**  Baseline value  Endpoint value  % change  P-value | 38.06  46.86  23.1  0.007 | 40.29  50.20  24.6  0.050 | p>0.05  P>0.05 |
| **SF-36 Mental, mean**  Baseline value  Endpoint value  % change  P-value | 46.56  53.37  14.6  0.125 | 56.64  57.62  1.73  0.373 | p>0.05  P>0.05 |
| **González-Lucena et al [6]** | **ACL reconstruction, n=8** | **Microfracture, n=8** | **P-value** |
| **Lysholm score, mean**  Endpoint | 86.6 | 90 | >0.05 among subgroups and total sample mean 88.6 |
| **LaPrade et al [11]** | MAT alone n = 19 | MAT and other procedures n=21, including ACL 10, microfracture 5, OCA 3, osteotomy 3 | No significant difference in outcomes |
| **Marcacci et al [15]** | MAT alone n=22 | MAT + n= 10. ACL 4, osteotomy 6 | No significant difference in outcomes |
| **Rue et al [23]** | **MAT+ACI, n=16 transplants** | **MAT+OA, n=15 transplants** |  |
| **Lyshom, mean (SD)**  Baseline value  Endpoint value  P-value | 55.0 (16.0)  79.4 (11.9)  <0.001 | 42.0 (14.5)  68.2 (21.3)  0.001 | P = 0.037 |
| **IKDC, mean (SD)**  Baseline value  Endpoint value  P-value | 45.5 (8.2)  76.0 (10.8)  <0.001 | 31.4 (12.8)  57.1 (17.8)  <0.001 | P = 0.002  P = 0.0024 |
| **Tegner, mean (SD)**  Baseline value  Endpoint value  P-value | 5.5 (2.9)  7.3 (1.5)  0.026 | 4.4 (3.7)  6.2 (2.9)  0.03 |  |
| **SF-12 Physical, mean (SD)**  Baseline value  Endpoint value  P-value | 40.6 (6.3)  45.6 (3.5)  0.009 | 37.0 (8.2)  42.2 (6.9)  0.081 |  |
| **SF-12 Mental, mean (SD)**  Baseline value  Endpoint value  % change  P-value | 58.2 (6.4)  54.7 (6.5)  -6.0  0.159 | 52.6 (11.3)  55.7 (9.9)  5.9  0.135 | p=0.038 |

^a^ For studies reporting Noyes and KOOS scores[5, 23] or Cincinnati knee rating scale[17, 19]

## Table S10: Combined procedures subgroups: survival

| **Noyes and Barber-Westin [17]** | **Concurrent osteochondral autograft, n=52 (transplants)** | **No concurrent osteochondral autograft, n=20 (transplants)** |  |
| --- | --- | --- | --- |
| **Survival %, mean (95% CI) at**  2 years  5 years  7 years  10 years  15 years | 88 (76 to 95)  78 (64 to 88)  76 (62 to 86)  55 (41 to 69)  19 (10 to 33) | 75 (50 to 90)  75 (50 to 90)  50 (28 to 72)  20 (7 to 44)  20 (7 to 44) | p<0.05 |
| **Parkinson et al [21]** | **N not reported** |  |  |
| HR for additional procedures versus isolated MAT | 1.62 (95% CI 0.31, 8.43) | p=0.56 |  |

## References

1. Abat F, Gelber PE, Erquicia JI, Tey M, Gonzalez-Lucena G, Monllau JC (2013) Prospective comparative study between two different fixation techniques in meniscal allograft transplantation. Knee Surg Sports Traumatol Arthrosc 21:1516-1522

2. Abrams GD, Hussey K, Harris JD, Cole BJ (2014) Combined Meniscus and Osteochondral Allograft Transplantation: Minimum Two-Year Follow-up with an Analysis of Failures. Orthop J Sports Med doi.org/10.1177/2325967114S00048

3. Abrams GD, Hussey KE, Harris JD, Cole BJ (2014) Clinical results of combined meniscus and femoral osteochondral allograft transplantation: minimum 2-year follow-up. Arthroscopy 30:964-970.e961

4. Carter T, Rabago M, Jordan S, Chu C, Macdonald P, Harner C, et al. (2012) Meniscal Allograft Transplantation: 10 Year Follow-up (SS-31). Arthroscopy 28:e17-e18

5. Cole BJ, Dennis MG, Lee SJ, Nho SJ, Kalsi RS, Hayden JK, et al. (2006) Prospective evaluation of allograft meniscus transplantation: a minimum 2-year follow-up. Am J Sports Med 34:919-927

6. Gonzalez-Lucena G, Gelber PE, Pelfort X, Tey M, Monllau JC (2010) Meniscal allograft transplantation without bone blocks: a 5- to 8-year follow-up of 33 patients. Arthroscopy 26:1633-1640

7. Kempshall PJ, Parkinson B, Thomas M, Robb C, Standell H, Getgood A, et al. (2015) Outcome of meniscal allograft transplantation related to articular cartilage status: advanced chondral damage should not be a contraindication. Knee Surg Sports Traumatol Arthrosc 23:280-289

8. Kim CW, Kim JM, Lee SH, Kim JH, Huang J, Kim KA, et al. (2011) Results of isolated lateral meniscus allograft transplantation: focus on objective evaluations with magnetic resonance imaging. Am J Sports Med 39:1960-1967

9. Kim JM, Bin SI, Lee BS, Kim NK, Song JH, Choi JW, et al. (2017) Long-term Survival Analysis of Meniscus Allograft Transplantation With Bone Fixation. Arthroscopy 33:387-393

10. Kim JM, Lee BS, Kim KH, Kim KA, Bin SI (2012) Results of meniscus allograft transplantation using bone fixation: 110 cases with objective evaluation. Am J Sports Med 40:1027-1034

11. LaPrade RF, Wills NJ, Spiridonov SI, Perkinson S (2010) A prospective outcomes study of meniscal allograft transplantation. Am J Sports Med 38:1804-1812

12. Lee BS, Bin SI, Kim JM (2016) Articular Cartilage Degenerates After Subtotal/Total Lateral Meniscectomy but Radiographic Arthrosis Progression Is Reduced After Meniscal Transplantation. Am J Sports Med 44:159-165

13. Lee DH, Kim SB, Kim TH, Cha EJ, Bin SI (2010) Midterm outcomes after meniscal allograft transplantation: comparison of cases with extrusion versus without extrusion. Am J Sports Med 38:247-254

14. Mahmoud A, Young J, Bullock-Saxton J, Myers P (2018) Meniscal Allograft Transplantation: The Effect of Cartilage Status on Survivorship and Clinical Outcome. Arthroscopy 34:1871-1876.e1871

15. Marcacci M, Zaffagnini S, Marcheggiani Muccioli GM, Grassi A, Bonanzinga T, Nitri M, et al. (2012) Meniscal allograft transplantation without bone plugs: a 3-year minimum follow-up study. Am J Sports Med 40:395-403

16. McCormick F, Harris JD, Abrams GD, Hussey KE, Wilson H, Frank R, et al. (2014) Survival and reoperation rates after meniscal allograft transplantation: analysis of failures for 172 consecutive transplants at a minimum 2-year follow-up. Am J Sports Med 42:892-897

17. Noyes FR, Barber-Westin SD (2016) Long-term Survivorship and Function of Meniscus Transplantation. Am J Sports Med 44:2330-2338

18. Noyes FR, Barber-Westin SD (2015) Meniscal Transplantation in Symptomatic Patients Under Fifty Years of Age: Survivorship Analysis. J Bone Joint Surg Am 97:1209-1219

19. Noyes FR, Barber-Westin SD, Rankin M (2004) Meniscal transplantation in symptomatic patients less than fifty years old. J Bone Joint Surg Am 86-A:1392-1404

20. Noyes FR, Barber-Westin SD, Rankin M (2005) Meniscal transplantation in symptomatic patients less than fifty years old. J Bone Joint Surg Am 87 Suppl 1:149-165

21. Parkinson B, Smith N, Asplin L, Thompson P, Spalding T (2016) Factors Predicting Meniscal Allograft Transplantation Failure. Orthop J Sports Med 4:2325967116663185.

22. Riboh JC, Tilton AK, Cvetanovich GL, Campbell KA, Cole BJ (2016) Meniscal Allograft Transplantation in the Adolescent Population. Arthroscopy 32:1133-1140.e1131

23. Rue JP, Yanke AB, Busam ML, McNickle AG, Cole BJ (2008) Prospective evaluation of concurrent meniscus transplantation and articular cartilage repair: minimum 2-year follow-up. Am J Sports Med 36:1770-1778

24. Saltzman BM, Bajaj S, Salata M, Daley EL, Strauss E, Verma N, et al. (2012) Prospective long-term evaluation of meniscal allograft transplantation procedure: a minimum of 7-year follow-up. J Knee Surg 25:165-175

25. Saltzman BM, Meyer MA, Leroux TS, Gilelis ME, Debot M, Yanke AB, et al. (2017) The Influence of Full-Thickness Chondral Defects on Outcomes Following Meniscal Allograft Transplantation: A Comparative Study. Arthroscopy 34:519-529

26. Saltzman BM, Meyer MA, Weber AE, Poland SG, Yanke AB, Cole BJ (2017) Prospective Clinical and Radiographic Outcomes After Concomitant Anterior Cruciate Ligament Reconstruction and Meniscal Allograft Transplantation at a Mean 5-Year Follow-up. Am J Sports Med 45:550-562

27. Stone KR, Adelson WS, Pelsis JR, Walgenbach AW, Turek TJ (2010) Long-term survival of concurrent meniscus allograft transplantation and repair of the articular cartilage: a prospective two- to 12-year follow-up report. J Bone Joint Surg Br 92:941-948

28. Stone KR, Pelsis J, Surrette S, Stavely A, Walgenbach AW (2013) Meniscus Allograft Transplantation Allows Return To Sporting Activities. Arthroscopy 29:e52-e53

29. Stone KR, Pelsis JR, Surrette ST, Walgenbach AW, Turek TJ (2015) Meniscus transplantation in an active population with moderate to severe cartilage damage. Knee Surg Sports Traumatol Arthrosc 23:251-257

30. Stone KR, Walgenbach AW, Turek TJ, Freyer A, Hill MD (2006) Meniscus allograft survival in patients with moderate to severe unicompartmental arthritis: a 2- to 7-year follow-up. Arthroscopy 22:469-478

31. van Arkel ER, de Boer HH (1995) Human meniscal transplantation. Preliminary results at 2 to 5-year follow-up. Journal of Bone & Joint Surgery - British Volume 77:589-595

32. van Arkel ER, de Boer HH (2002) Survival analysis of human meniscal transplantations. J Bone Joint Surg Br 84:227-231

33. Van Der Straeten C, Byttebier P, Eeckhoudt A, Victor J (2016) Meniscal Allograft Transplantation Does Not Prevent or Delay Progression of Knee Osteoarthritis. PLoS ONE 11:e0156183

34. van der Wal RJ, Thomassen BJ, van Arkel ER (2009) Long-term clinical outcome of open meniscal allograft transplantation. Am J Sports Med 37:2134-2139

35. Verdonk PC, Demurie A, Almqvist KF, Veys EM, Verbruggen G, Verdonk R (2005) Transplantation of viable meniscal allograft. Survivorship analysis and clinical outcome of one hundred cases. J Bone Joint Surg Am 87:715-724

36. Verdonk PC, Verstraete KL, Almqvist KF, De Cuyper K, Veys EM, Verbruggen G, et al. (2006) Meniscal allograft transplantation: long-term clinical results with radiological and magnetic resonance imaging correlations. Knee Surg Sports Traumatol Arthrosc 14:694-706

## Appendix 1: Literature searches

The figures below are for the full allografts review.

Ovid Medline Search strategy

1. exp Allografts/

2. allograft*.mp.

3. 1 or 2

4. (osteochondral or cartilage or chondrocyte* or osteoarticular or chondral or articular or condyle or tibia* or knee* or patell* or menisc* or ligament* or femoral or femur or patellofemoral).tw.

5. exp Cartilage, Articular/su [Surgery]

6. exp Cartilage/tr [Transplantation]

7. chondrocytes/tr [Transplantation]

8. Knee/su [Surgery]

9. exp Knee Joint/su [Surgery]

10. exp Ligaments, Articular/su [Surgery]

11. exp Menisci, Tibial/su, tr [Surgery, Transplantation]

12. 4 or 5 or 6 or 7 or 8 or 9 or 10 or 11

13. 3 and 12

14. (letter or editorial).pt.

15. 13 not 14

16. limit 15 to english language

17. limit 16 to yr="2000 -Current"

The Ovid Medline search strategy above was adapted as appropriate for Ovid Embase, Web of Science and the Cochrane Library. The searches were last run on February 15^th^, 2018.

All records were downloaded into the bibliographic database Endnote. After deduplication, 5013 articles were screened by one reviewer for obvious exclusions. The title and abstracts of the remaining 3468 articles were screened independently by two reviewers. The full texts of 815 articles were then obtained for further scrutiny.

The search was designed to be very sensitive in order to include all study designs and to retrieve articles for clinical effectiveness, costs and economics, natural history and prognosis. The searches included articles published as full text and conference abstracts, and were limited to English language articles only. The reference lists of systematic reviews and included studies were also checked.
